# Global prevalence and risk factors of osteoporosis in patients with chronic obstructive pulmonary disease: a systematic review and meta-analysis

**DOI:** 10.1186/s12889-026-28811-1

**Published:** 2026-08-19

**Authors:** Chunhui Yu, Xuejiao Teng, Weilong Du, Xilan Ma, Chunhao Yu, Yunfeng Xia, Jipeng Hui, Fei Xie, Ji Li, Shilong Hu

**Affiliations:** 1 The First Clinical College, Liaoning University of Traditional Chinese Medicine, Shenyang, Liaoning Province China; 2 The Third Department of Pulmonary Diseases, The Affiliated Hospital of Liaoning University of Traditional Chinese Medicine, Shenyang, Liaoning Province China; 3 The Third Department of Orthopedics, The Second Affiliated Hospital of Liaoning University of Traditional Chinese Medicine, Shenyang, Liaoning Province China; 4 Second Department of Gastroenterology, Northeast International Hospital Houde Campus, Shenyang, Liaoning Province China; 5 The Center for Preventive Treatment of Disease, The Second Affiliated Hospital of Liaoning University of Traditional Chinese Medicine, Shenyang, Liaoning Province China; 6 College of Bioscience and Biotechnology, Hunan Agricultural University, Changsha, Hunan Province China; 7 Department of Andrology, The Affiliated Hospital of Liaoning University of Traditional Chinese Medicine, Shenyang, Liaoning Province China

**Keywords:** Chronic obstructive pulmonary disease, Osteoporosis, Prevalence, Risk factors, Systematic review, Meta-analysis, Public health

## Abstract

**Background:**

Osteoporosis (OP) is a common comorbidity in patients with chronic obstructive pulmonary disease (COPD), but reported prevalence estimates vary substantially across studies, and the magnitudes of associations between OP and potentially modifiable factors remain uncertain. This systematic review and meta-analysis aimed to provide an updated estimate of the prevalence of OP among patients with COPD, explore potential sources of heterogeneity, and synthesise adjusted associations with selected clinical factors.

**Methods:**

EMBASE, CINAHL, Scopus, PubMed, and the Cochrane Library were searched from inception to 5 February 2026. Only observational studies published in English involving adults with objectively diagnosed COPD and reporting OP prevalence or associated factors were included. Two reviewers independently conducted study selection, data extraction, and quality assessment using the Newcastle–Ottawa Scale or the AHRQ assessment tool. Random-effects models were used for all quantitative syntheses. Risk-factor analyses were based exclusively on adjusted ORs. Subgroup analyses, leave-one-out sensitivity analyses, and univariable meta-regression were used to explore heterogeneity.

**Results:**

Eighty-seven studies involving 1,453,879 patients with COPD were included. The pooled prevalence of OP was 33.8% (95% CI 31.4%–36.1%), with extremely high between-study heterogeneity (I²=99.7%). Prevalence estimates varied geographically, ranging from 28.7% in Europe and 28.8% in Africa to 42.3% in North America. The methodological quality score was significantly associated with variation in prevalence estimates in univariable meta-regression (*P* < 0.001), although substantial residual heterogeneity remained. Underweight was associated with higher odds of OP (OR = 3.91, 95% CI 1.36–11.24). Corticosteroid exposure was also associated with OP (OR = 2.85, 95% CI 1.45–5.59). In an exploratory analysis based on two studies involving 172 participants, cumulative systemic corticosteroid exposure exceeding 1,000 mg prednisone-equivalent was associated with higher odds of OP (OR = 7.36, 95% CI 1.70–31.98). Higher FEV₁% predicted was associated with lower odds of OP (OR = 0.97, 95% CI 0.96–0.98). No statistically significant associations were identified for age ≥ 65 years or overweight status.

**Conclusions:**

OP affects approximately one-third of patients with COPD across the included studies. Underweight, corticosteroid exposure, and reduced lung function were associated with OP, but the findings should be interpreted cautiously because of extreme residual heterogeneity, differences in covariate adjustment, and limited evidence available for some subgroup analyses. Standardised diagnostic methods and better reporting of clinical, treatment-related, and sociodemographic characteristics are needed to improve future risk estimation.

**PROSPERO registration number:**

CRD420261381268.

**Supplementary Information:**

The online version contains supplementary material available at https://doi.org/10.1186/s12889-026-28811-1.

## Introduction

Chronic obstructive pulmonary disease (COPD) is a prevalent, preventable, and treatable condition characterised by persistent respiratory symptoms and airflow limitation that is not fully reversible. This limitation is typically progressive and closely associated with an exaggerated chronic inflammatory response of the airways and lung parenchyma to noxious particles or gases. Acute exacerbations and comorbid conditions substantially influence disease severity and clinical outcomes in individual patients [1]. Global burden of disease estimates indicate that COPD affects over 200 million people and remains the third leading cause of death worldwide [2]. Osteoporosis (OP) is a systemic skeletal disorder characterised by reduced bone mass, deterioration of bone microarchitecture, and increased skeletal fragility, which together result in a markedly elevated risk of fracture. Its pathophysiology is multifactorial, involving both reduced bone formation and accelerated bone resorption [3, 4]. As highly prevalent chronic conditions, COPD and OP substantially impair patients’ quality of life and impose considerable socioeconomic burdens. Their coexistence further exacerbates functional decline and is associated with increased healthcare utilisation and mortality [5].

The association between COPD and OP is biologically plausible and increasingly framed within the concept of a “lung–bone axis” [6]. Chronic systemic inflammation represents a key mechanistic link [7, 8]. Persistently elevated levels of pro-inflammatory cytokines in COPD promote osteoclast activation while suppressing osteoblast function, resulting in imbalanced bone remodelling and progressive bone loss [9, 10]. This inflammatory milieu not only drives lung function decline but also adversely affects skeletal integrity, increasing the risk of OP and fragility fractures [11, 12].

In addition, the pharmacological management of COPD is an important contributor to skeletal risk. Systemic corticosteroids are a well-established iatrogenic cause of secondary OP when used long term because they inhibit bone formation and stimulate bone resorption [13, 14].

Previous evidence syntheses have established that OP is a common comorbidity in patients with COPD, but important uncertainties remain. Chen et al. [15] conducted a systematic review and meta-analysis of 58 studies involving 8,753 patients with COPD and reported a pooled OP prevalence of approximately 38%, while also identifying low BMI and reduced muscle mass as important associated factors. That review also explored heterogeneity according to several population- and study-level characteristics, including age, sex, sample size, COPD severity, recruitment setting, and geographical region. However, the potential contribution of other methodological characteristics, including methodological quality, study design, and BMD assessment methods, was not systematically quantified, and the epidemiological evidence base has expanded considerably since that review was conducted.

Penedones et al. [16] subsequently conducted a scoping review of the relationship between COPD and OP and highlighted substantial methodological heterogeneity across study populations, diagnostic approaches, and outcome definitions. However, owing to its descriptive scoping-review design, that study did not provide an updated pooled prevalence estimate or quantitatively evaluate the contribution of different study-level characteristics to between-study heterogeneity. More recently, Huang et al. [17] focused specifically on the association between long-term inhaled corticosteroid (ICS) exposure and OP or fracture risk in patients with COPD and explored selected potential contributors to heterogeneity. However, its scope was centred on ICS-related skeletal outcomes rather than the overall epidemiological burden of OP or the broader spectrum of clinical factors associated with OP among patients with COPD.

Consequently, several questions remain only partly resolved. Reported prevalence estimates vary considerably across studies, potentially because of differences in geographical settings, study designs, participant characteristics, OP diagnostic methods, skeletal measurement sites, COPD severity, and methodological quality. The extent to which these study-level characteristics contribute to the marked variation in reported prevalence estimates remains uncertain. Uncertainty also persists regarding the magnitude of associations between OP and clinically relevant factors in patients with COPD. Although low body weight, corticosteroid exposure, impaired lung function, older age, and sex have frequently been investigated, the reported associations are inconsistent, and differences in exposure definitions, diagnostic procedures, and covariate adjustment complicate their interpretation. In particular, corticosteroid exposure is often reported without detailed stratification by route of administration or cumulative dose, thereby limiting the assessment of potentially clinically relevant exposure patterns.

Against this background, this systematic review and meta-analysis aimed to provide an updated quantitative synthesis of the prevalence of OP among patients with COPD and to build on previous evidence by examining potential sources of between-study heterogeneity and selected clinically relevant factors associated with OP. Specifically, we sought to (1) estimate the overall prevalence of OP and compare prevalence estimates across geographical regions, study designs, BMI and age categories, and BMD assessment methods; (2) explore study-level contributors to heterogeneity using subgroup analyses and univariable meta-regression; and (3) synthesise adjusted associations between OP and selected clinical factors, including sex, BMI, corticosteroid exposure, FEV₁% predicted, and age. By addressing these objectives, this study aimed to provide a more comprehensive and up-to-date assessment of the current evidence, clarify potential methodological contributors to the substantial variation in reported prevalence estimates, and offer a more granular evaluation of selected clinically relevant factors, with particular attention to corticosteroid exposure where the available data permitted such evaluation.

## Materials and methods

### Literature search

A systematic search of the EMBASE, CINAHL, Scopus, PubMed and Cochrane Library databases was conducted using a strategy that combined subject headings and free-text terms. The databases were searched up to 5 February 2026, and the specific search terms are detailed in Supplementary Material Method 1. This systematic review and meta-analysis was prospectively registered on the PROSPERO platform (Registration number: CRD420261381268; Registration URL: https://www.crd.york.ac.uk/PROSPERO/view/CRD420261381268). The detailed search strategy is provided in Supplementary Method 1.

## Inclusion and exclusion criteria

### Inclusion criteria

#### Studies were included if they met all of the following criteria

##### Study population

The study population comprised adults aged ≥ 18 years with COPD. COPD had to be identified using spirometry, an explicitly documented clinical diagnosis based on established criteria, or, for large healthcare databases, a clearly specified and verifiable administrative case definition or diagnostic coding algorithm. Studies relying solely on participant self-report were not eligible.

##### Study outcomes

The study reported the prevalence of OP or factors associated with OP among patients with COPD. OP was required to be identified using clearly defined diagnostic criteria, including BMD-based methods such as dual-energy X-ray absorptiometry (DXA), quantitative computed tomography (QCT), or quantitative ultrasound (QUS), or an explicitly documented clinical or administrative diagnosis based on a prespecified and verifiable case definition, including diagnostic coding algorithms. Where BMD was measured, established diagnostic thresholds, such as a T-score of ≤ − 2.5, were required.

##### Study design

Eligible studies were observational studies, including cross-sectional, case-control, and cohort studies.

##### Language

Only studies published in English were included.

## Exclusion criteria

### Studies were excluded if they met any of the following criteria

#### Insufficiently defined diagnostic methods

COPD or OP was identified solely through participant self-report, non-standardised subjective judgement, or medical-record or administrative data without a clearly specified and verifiable case definition.

#### Ineligible publication type

Animal studies, in vitro studies, narrative reviews, systematic reviews, meta-analyses, case reports, conference abstracts, commentaries, editorials, letters, and publications for which the full text was unavailable were excluded.

### Operational clarifications

The registered PROSPERO record prespecified the inclusion of patients diagnosed with COPD and observational studies reporting OP prevalence or associated factors, but did not explicitly enumerate all acceptable diagnostic modalities. The detailed diagnostic definitions described above represent operational clarifications of the original eligibility criteria, introduced to ensure consistent application across different observational study designs and data sources. These clarifications did not change the underlying target population or outcomes specified in the registered review.

### Data extraction and quality assessment

Two reviewers (YCH and TXJ) independently screened the literature and used a standardised form to extract data on the first author, year of publication, diagnostic criteria, study population, study design, sample size, and factors associated with OP. The extracted data were cross-checked, and disagreements were resolved through discussion with the corresponding author (HSL).

Two reviewers independently assessed the methodological quality of the included studies. Cohort and case-control studies were evaluated using the Newcastle–Ottawa Scale (NOS), which assesses study quality across the domains of selection, comparability, and assessment of exposure or outcome, with a maximum score of 9 stars. Studies scoring ≥ 7, 5–6, and ≤ 4 stars were classified as high, moderate, and low quality, respectively. Cross-sectional studies were assessed using the 11-item Agency for Healthcare Research and Quality (AHRQ) checklist. Each item rated “yes” was assigned 1 point, whereas “no” or “unclear” was assigned 0 points. Studies scoring ≥ 8, 6–7, and ≤ 5 points were classified as high, moderate, and low quality, respectively.

For the risk-factor analyses, only adjusted ORs reported by the primary studies were extracted. Factors reported in at least two studies were considered eligible for quantitative synthesis. Unadjusted effect estimates were not included in the pooled analyses.

### Statistical methods

Stata 19.0 was used for all statistical analyses. Clinical and methodological diversity was anticipated across the included observational studies because of differences in study populations, geographical settings, study designs, OP diagnostic methods, and exposure and risk-factor definitions. Accordingly, a random-effects model was used for all quantitative syntheses, irrespective of the magnitude or statistical significance of heterogeneity. Pooled prevalence estimates and ORs were calculated with their corresponding 95% CIs.

Between-study heterogeneity was assessed using Cochran’s Q test and the I² statistic. These measures were used to describe and quantify heterogeneity rather than to determine the choice of statistical model. Subgroup analyses and univariable meta-regression were conducted to explore potential sources of heterogeneity. Leave-one-out sensitivity analyses were performed to evaluate the robustness of the pooled estimates. When a sufficient number of studies were available, publication bias was assessed using funnel plots and Egger’s test. A two-sided P-value < 0.05 was considered statistically significant, except for Cochran’s Q test, for which *P* < 0.10 was considered indicative of potential statistical heterogeneity.

All pooled analyses of factors associated with OP were based exclusively on adjusted ORs. Therefore, adjusted and unadjusted estimates were not combined, and separate analyses stratified by adjustment status were not applicable.

## Results

### Literature search results

**Fig. 1:**
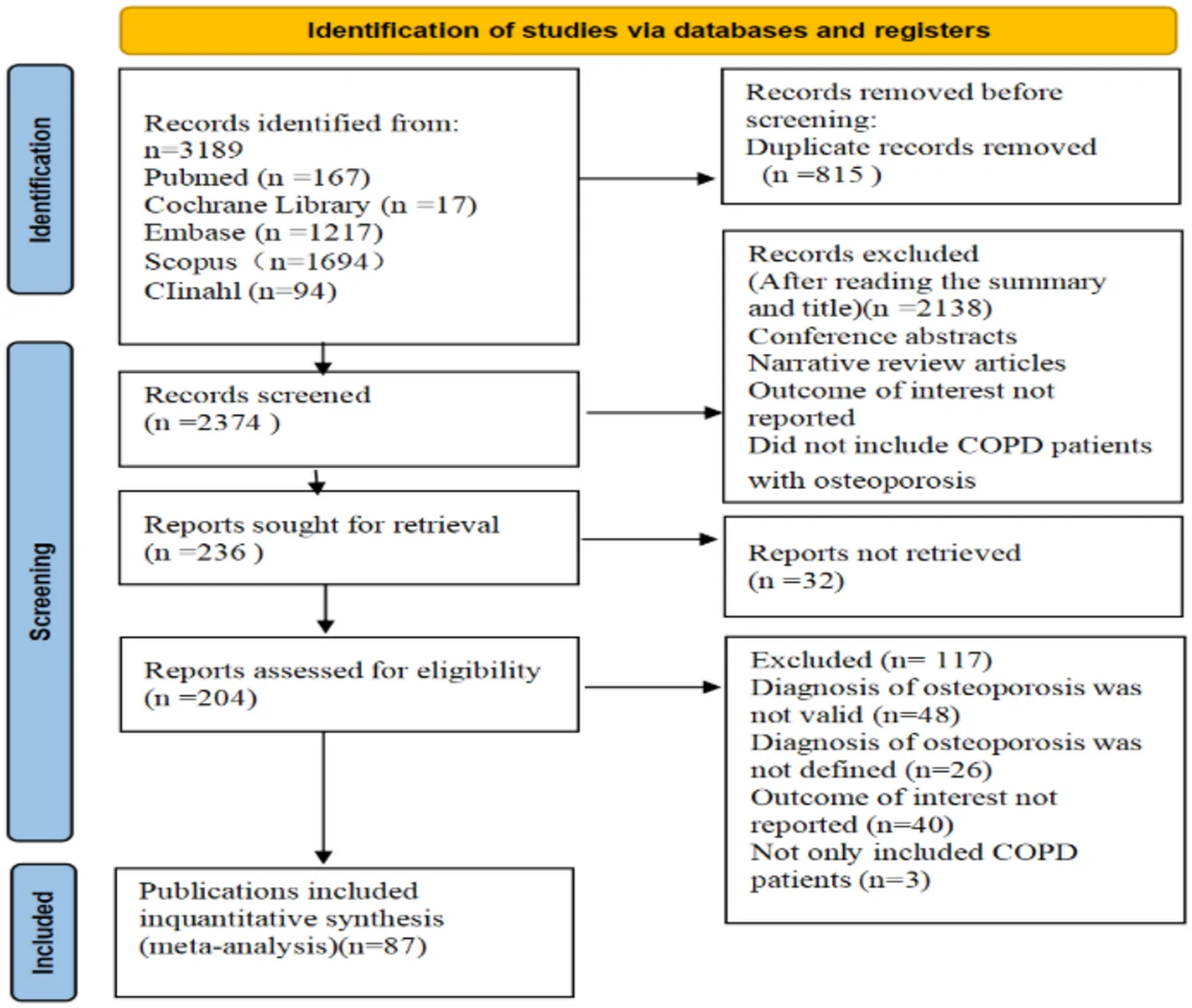
The process of article inclusion

The initial search yielded 3,189 articles. After 815 duplicates had been removed, a further 2,138 articles were excluded following title and abstract screening. The full texts of 32 articles were unavailable, and a further 117 articles were excluded after full-text review. Ultimately, 87 articles were included in the analysis. The inclusion process is illustrated in Fig. 1, and a summary of the included articles is provided in Supplementary Table S1.

### Characteristics of included studies and quality assessment

The review included 87 studies: 73 cross-sectional studies, 5 case-control studies, and 9 cohort studies. Based on the predefined quality assessment criteria, 24 studies were classified as high quality, 30 as moderate quality, and 33 as low quality. Detailed results of the quality assessment are provided in Supplementary Tables S2–S3.

### Meta-analysis results

#### The prevalence of OP among individuals with COPD

Overall prevalence: A total of 87 articles [18–104] reported the overall prevalence of concomitant OP among 1,453,879 participants with COPD. There was considerable heterogeneity across the studies (I² = 99.7%, *P* < 0.05), and a random-effects model was employed. The pooled prevalence was 33.80% [95% CI (31.4%, 36.1%), *P* < 0.01], with a statistically significant difference. The results are presented in Supplementary Figure S1 and Table 1.

First, a subgroup analysis was conducted according to the continent in which each study population was located. This subgroup analysis included all 87 studies [18–104]. Prevalence estimates were pooled for Asia, Europe, the Americas, Oceania, and Africa according to the geographical location of the study populations. Differences in prevalence across continents were compared, whilst heterogeneity within each subgroup was assessed to determine whether geographical location influenced the prevalence of OP among patients with COPD. Forty-one articles [18, 20, 25, 30, 31, 41–43, 46–51, 53–56, 58, 59, 61, 62, 66, 68, 73–75, 78–80, 83, 84, 86, 90–92, 95–97, 100, 104] reported the prevalence of OP in patients with COPD in Asia, involving a total of 1,357,838 participants. There was considerable heterogeneity across the studies (I² = 97.1%, *P* < 0.05), and a random-effects model was employed. The results showed a prevalence of 32.90% [95% CI (31.0%, 34.9%), *P* < 0.01], with the difference being statistically significant. Thirty-two articles [19, 21–23, 26, 28, 29, 32, 33, 36–40, 44, 45, 57, 60, 63–65, 69, 70, 72, 76, 82, 93, 94, 98, 101–103] reported the prevalence of COPD complicated by OP in Europe, involving a total of 94,981 participants. There was considerable heterogeneity across the studies (I² = 98.2%, *P* < 0.05), and a random-effects model was employed. The results showed a prevalence of 28.70% [95% CI (25.3%, 32.2%), *P* < 0.01], with the difference being statistically significant. Four articles [24, 27, 52, 71] reported on the prevalence of COPD combined with OP in North America, involving a total of 313 participants. There was considerable heterogeneity among the studies (I² = 92.8%, *P* < 0.05), and a random-effects model was employed. The results showed a prevalence of 42.30% [95% CI (20.4%, 64.2%), *P* < 0.01], with a statistically significant difference. Eight articles [34, 35, 67, 81, 85, 87–89] reported the prevalence of COPD with OP in South America, involving a total of 647 participants. There was considerable heterogeneity among the studies (I² = 57.2%, *P* = 0.022), and a random-effects model was employed. The results showed a prevalence of 38.30% [95% CI (32.5%, 44.0%), *P* < 0.01], indicating a statistically significant difference. Two articles [77, 99] reported the prevalence of OP among 100 participants with COPD in Africa. Given the low heterogeneity (I² = 0.0%, *P* = 0.508), a random-effects model was employed. The results showed a prevalence of 28.80% [95% CI (20.0%, 37.7%), *P* < 0.01], indicating a statistically significant difference.

Overall, the prevalence of OP among patients with COPD varied little across Europe, Asia, and Africa, whilst estimates for the Americas were slightly above the overall average. However, the sample size for the Americas was limited, with only 12 studies and 960 participants, which reduced the representativeness of these data. This finding should therefore be interpreted with caution. Future large-scale studies with more balanced geographical representation are needed to validate these findings. A summary of the subgroup analyses for each region is presented in Supplementary Figure S1.

Secondly, a subgroup analysis was conducted based on the study design of the included articles. This subgroup analysis included all 87 studies [18–104], categorised according to study design (cross-sectional, case-control and cohort studies). The prevalence of OP among patients with COPD was pooled separately for each study type to compare estimates across study designs. Heterogeneity within each subgroup was also assessed to determine whether study design influenced the pooled effect size or contributed to heterogeneity. Seventy-three cross-sectional studies [18–38, 42–72, 74–78, 80, 84–88, 90–94, 97, 98, 100, 101, 104] investigated the prevalence of COPD with co-morbid osteoporosis, involving a total of 1,315,629 participants, with considerable heterogeneity across the studies (I² = 96.2%, *P* < 0.05). A random-effects model was employed, and the results indicated a prevalence of 33.70% [95% CI (31.5%, 36.0%), *P* < 0.01], representing a statistically significant difference. Five case-control studies [40, 41, 73, 81, 99] investigated the prevalence of COPD complicated by osteoporosis, involving a total of 330 participants, with considerable heterogeneity across the studies (I² = 91.8%, *P* < 0.05). A random-effects model was applied, and the results showed a prevalence of 49.8% [95% CI (32.0%, 67.5%), *P* < 0.01], indicating a statistically significant difference. Nine cohort studies [39, 79, 82, 83, 89, 95, 96, 102, 103] investigated the prevalence of COPD complicated by osteoporosis, involving a total of 137,920 participants, with considerable heterogeneity across the studies (I² = 99.9%, *P* < 0.05). A random-effects model was applied, and the results showed a prevalence of 24.60% [95% CI (15.8%, 33.4%), *P* < 0.01], indicating a statistically significant difference. Although point estimates differed across study designs, with case-control studies showing a higher prevalence and wider confidence intervals, possibly because of inherent selection bias and smaller sample sizes, the prevalence in cross-sectional studies was closest to the overall pooled estimate. Estimates from cohort studies, however, remain unstable due to their limited number and extremely high heterogeneity. Overall, study design has some influence on the effect size, although it is not the sole source of heterogeneity in this study. Furthermore, results across all subgroups indicate a significant burden of OP among patients with COPD, and this finding is consistent across study designs. The results are shown in Supplementary Figure S2 and Table 1.

**Table 1:** The overall prevalence of COPD with OP and the corresponding subgroup analysis by continent are presented

Subgroup Name	Number of individuals with OP	Total SampleSize(*n*)	Detection rates/(95%CI)(%)	*P* Value	I2(%)	Effect Model	Egger
Total detection rate	298,141	1,453,879	33.80(31.4, 36.1)	*P*<0.01	99.7%	random	*P* < 0.001
continent							
Asia	290,529	1,357,838	32.90(31.0, 34.9)	*P*<0.01	97.1%	random	
Europe	7170	94,981	28.70(25.3, 32.2)	*P*<0.01	98.2%	random	
North America	162	313	42.30(20.4, 64.2)	*P*<0.01	92.8%	random	
South America	251	647	38.30(32.5, 44.0)	*P*<0.01	57.2%	random	
Africa	29	100	28.80(20.0, 37.7)	*P*<0.01	0.0%	random	
Type of English Literature							
cross-sectional study	279,340	1,315,629	33.70(31.5, 36.0)	*P*<0.01	96.2%	random	
cohort study	18,646	137,920	24.60(15.8, 33.4)	*P*<0.01	99.9%	random	
case-control study	155	330	49.8(32.0, 67.5)	*P*<0.01	91.8%	random	

Subgroup analyses based on continent revealed significant geographical variations in the prevalence of COPD with OP. To further investigate the influence of population characteristics on the prevalence of comorbid OP, subgroup analyses stratified by BMI and age were also conducted. According to the WHO international BMI classification criteria, the BMI cut-off points are as follows: underweight (BMI < 18.5 kg/m²), normal weight (18.5–24.9 kg/m²), overweight (25.0–29.9 kg/m²), and obese (BMI ≥ 30.0 kg/m²) [105]. Subgroup analyses were planned for these four categories. Because the included studies reported insufficient raw data, none provided stratified data for underweight or obese populations; usable data could be extracted only for the normal-weight and overweight groups. Consequently, this study conducted subsequent subgroup analyses only for these two groups. Of the 87 included studies, only 69 [18, 21–23, 25–31, 33–38, 40, 42, 44, 46–56, 59, 60, 62–69, 71–79, 81, 83–89, 92–95, 97–100, 102–104] reported data for the normal-weight and overweight groups.

Of these, 37 articles [18, 21, 23, 25, 28, 30, 36, 37, 42, 46–51, 53–55, 59, 62, 66, 68, 69, 73–75, 77–79, 83, 84, 86, 92, 95, 97–99] reported the prevalence of OP among normal-weight patients with COPD. The total sample comprised 8,063 participants. Considerable between-study heterogeneity was observed (I² = 95.3%, *P* < 0.05), and a random-effects model was employed. The results showed a prevalence of 34.1% [95% CI (29.7%, 38.5%), *P* < 0.01], with the difference being statistically significant. Thirty-two articles [22, 26, 27, 29, 31, 33–35, 38, 40, 44, 52, 56, 60, 63–65, 67, 71, 72, 76, 81, 85, 87–89, 93, 94, 100, 102–104] reported the prevalence of OP among overweight individuals with COPD, involving a total of 10,316 participants. There was considerable heterogeneity across the studies (I² = 95.9%, *P* < 0.05), and a random-effects model was employed. The results showed a prevalence of 33.70% [95% CI (29.4%, 38.0%), *P* < 0.01], with a statistically significant difference; the results are shown in Supplementary Figure S3 and Table 2.

In summary, the pooled prevalence estimates of OP were numerically similar in normal-weight and overweight patients with COPD (34.1% vs. 33.7%), with substantially overlapping CIs. However, because these estimates were derived from different sets of studies rather than from direct within-study comparisons, no conclusion regarding the association between overweight status and OP can be drawn from this subgroup analysis. The association between overweight status and OP should instead be interpreted on the basis of the pooled adjusted OR analysis.

To examine the potential influence of age on the prevalence of OP among patients with COPD, we conducted an age-stratified subgroup analysis. Of the 87 included studies, 82 [18, 20–42, 44–60, 62–69, 71–100, 102–104] provided sufficient age-related data. Using 65 years as the cut-off, the study populations were classified into subgroups aged < 65 years and ≥ 65 years, and the pooled prevalence of OP was estimated separately for each subgroup.

Twenty-five studies [23, 24, 27, 28, 30, 31, 37, 41, 45, 51–53, 60, 64, 65, 69, 72, 77, 92, 96, 98–100, 102, 103], involving 59,618 participants, were included in the age < 65 years subgroup. Substantial between-study heterogeneity was observed (I²=96.6%, *P* < 0.05), and a random-effects model was applied. The pooled prevalence of OP was 32.7% (95% CI 28.7%–36.7%).

Fifty-seven studies [18, 20–22, 25, 26, 29, 32–36, 38–40, 42, 44, 46–50, 54–59, 62, 63, 66–68, 71, 73–76, 78–91, 93–95, 97, 104], involving 1,392,361 participants, were included in the age ≥ 65 years subgroup. Substantial between-study heterogeneity was also observed (I²=99.8%, *P* < 0.05), and a random-effects model was applied. The pooled prevalence of OP was 33.2% (95% CI 30.1%–36.3%). The results are presented in Supplementary Figure S4 and Table 2.

Overall, the pooled prevalence estimate was numerically slightly higher in the age ≥ 65 years subgroup than in the age < 65 years subgroup (33.2% vs. 32.7%). However, the absolute difference was small and the 95% CIs overlapped substantially, indicating no clear difference between the two subgroups. The weak numerical increase with age appeared less pronounced than might be expected given the established association between ageing and OP. One possible explanation is that older patients may be more likely to receive preventive interventions, including BMD assessment, calcium or vitamin D supplementation, and anti-resorptive treatment, which may partly attenuate age-related bone loss. However, this explanation remains speculative because baseline treatment information was inconsistently reported and could not be quantitatively evaluated. Future studies should report preventive and anti-OP treatment status more consistently to allow a more accurate assessment of the association between age and OP prevalence.

A subgroup analysis based on bone density measurement method included 81 studies [18–79, 81, 83–92, 95, 97–100, 102–104], which were categorised into two groups according to the diagnostic method used: DXA and non-DXA. Of these, six studies [20, 27, 61, 72, 83, 102] employed non-DXA assessment methods (including quantitative computed tomography [QCT] and quantitative ultrasound [QUS]) to evaluate the prevalence of COPD with OP. These studies included 1,081 participants and showed considerable between-study heterogeneity (I² = 97.5%, *P* < 0.05); a random-effects model was therefore applied. The results showed a prevalence of 26.20% [95% CI (10.1%, 42.4%), *P* < 0.01], with the difference being statistically significant. Seventy-five studies [18, 19, 21–26, 28–60, 62–71, 73–79, 81, 84–92, 95, 97–100, 103, 104] used DXA-based assessment criteria, involving a total of 15,633 participants. Given the high level of heterogeneity between studies (I² = 96.5%, *P* < 0.05), a random-effects model was applied. The results showed a prevalence of 36.70% [95% CI (33.2%, 40.1%), *P* < 0.01], with the difference being statistically significant. The results are presented in Supplementary Figure S5 and Table 2.

**Table 2:** The prevalence of OP among patients with COPD, stratified by BMI, age, and BMD measurement method, is presented

Subgroup Name	Number of individuals with OP	Total SampleSize(*n*)	Detection rates/(95%CI)(%)	*P* Value	I2(%)	Effect Model
BMI						
BMI Normal weight	2129	8063	34.10(29.7,38.5)	*P*<0.01	95.3%	random
BMI Overweight	2092	10,316	33.70(29.4,38.0)	*P*<0.01	95.9%	random
Age						
Age<65	14,630	59,618	32.70(28.7,36.7)	*P*<0.01	96.6%	random
Age ≥ 65	283,064	1,392,361	33.20(30.1,36.3)	*P*<0.01	99.8%	random
BMD assessment method						
DXA	3760	15,633	36.7(33.2, 40.1)	*P*<0.01	96.5%	random
non-DXA	306	1081	26.2(10.1, 42.4)	*P*<0.01	97.5%	random

The pooled prevalence estimate was numerically higher in studies using DXA than in those using non-DXA methods (36.7% vs. 26.2%). However, the CIs were wide and overlapped, and BMD assessment method was not significantly associated with variation in prevalence estimates in the univariable meta-regression (*P* = 0.128). Therefore, this subgroup difference should be interpreted as exploratory rather than as evidence that BMD assessment method is a confirmed source of heterogeneity. Differences in study populations, diagnostic thresholds, skeletal measurement sites, and other methodological characteristics may also have contributed to the observed numerical difference.

### Sensitivity analysis of the overall prevalence of COPD with OP

Given the extremely high heterogeneity in the overall prevalence estimate (I²=99.7%), leave-one-out sensitivity analyses were performed to assess the influence of individual studies. The magnitude of heterogeneity decreased after the studies by Liao et al. (2024) [80] and Akyea et al. (2019) [82] were excluded, indicating that these large database-based studies had a substantial influence on the pooled analysis. Their large sample sizes and broad population coverage may have introduced additional clinical and contextual variability. However, considerable heterogeneity remained after their exclusion, indicating that no single study fully accounted for the observed variability. Differences in study design, participant characteristics, OP diagnostic methods, skeletal measurement sites, COPD severity, medication exposure, and other unmeasured factors may also have contributed to the residual heterogeneity.

**Fig. 2:**
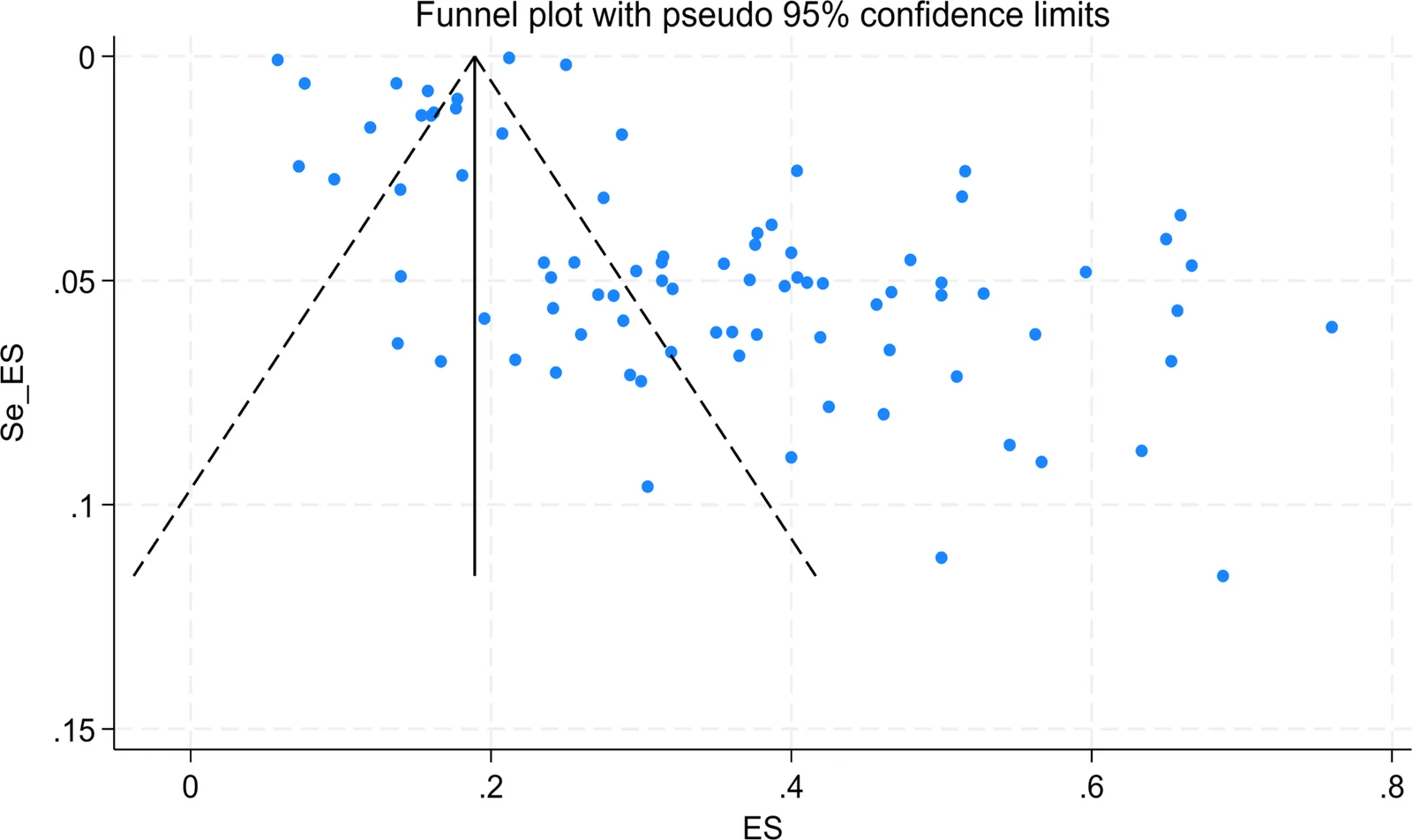
Funnel plot: The overall prevalence of COPD with OP

Potential publication bias and small-study effects were assessed using a funnel plot. Visual inspection suggested asymmetry, and Egger’s test was statistically significant (*P* < 0.001), indicating potential publication bias or small-study effects. Therefore, the pooled prevalence may have been overestimated. The results are shown in Fig. 2.

### Meta-regression analysis

In this meta-analysis, the heterogeneity in the pooled overall prevalence of OP among patients with COPD was extremely high (I² = 99.7 per cent); reliance solely on traditional subgroup analyses based on geographical location, BMI, age, study type, and bone density measurement method was insufficient to quantify fully the contribution of each factor to this heterogeneity. To identify the key drivers of heterogeneity more accurately, seven study-level characteristics were screened across all included studies, and univariable meta-regression analyses were conducted with pooled prevalence as the dependent variable. Because some studies did not fully report data on mean age or bone density measurement method, the final numbers of studies included in the respective regression models were 82 and 81.

Univariable meta-regression showed that study design (*P* = 0.514) and mean participant age (*P* = 0.715) were not significantly associated with variation in the prevalence estimates. Similarly, sample size (*P* = 0.324) and continent (*P* = 0.822) were not significantly associated with the pooled prevalence. The methodological quality score was significantly associated with variation in prevalence estimates (*P* < 0.001), whereas publication year showed a possible trend towards an association (*P* = 0.080). BMD assessment method was not statistically significant (*P* = 0.128), although the subgroup prevalence estimate was numerically higher in studies using DXA than in those using non-DXA methods.

These findings suggest that methodological quality may partly account for the observed heterogeneity. However, substantial residual heterogeneity remained, indicating that additional unmeasured clinical, demographic, treatment-related, and contextual characteristics may also contribute to between-study variation. Given that these analyses were univariable and based on aggregate study-level data, the findings should be interpreted as exploratory associations rather than evidence of independent or causal sources of heterogeneity. The results are presented in Supplementary Figs. 6–12.

Mean participant age was additionally included as a continuous study-level covariate in response to concerns regarding the broad age categorisation; however, it was not significantly associated with variation in prevalence estimates (*P* = 0.715). We also assessed the feasibility of incorporating other clinically relevant covariates. Sex distribution, COPD severity, corticosteroid exposure, urban versus rural setting, and country-level healthcare characteristics were either incompletely reported or defined using substantially different measures across the included studies. Therefore, these variables could not be incorporated into reliable meta-regression models without introducing substantial missing data, misclassification, and ecological bias.

### Risk factors for OP in COPD

We conducted meta-analyses of factors associated with OP among patients with COPD. Only factors reported by at least two studies were quantitatively synthesised, and all pooled estimates were based on adjusted ORs reported by the primary studies.

### Sex

Seven studies [36, 43, 44, 51, 58, 86, 87] assessed the association between sex and OP among patients with COPD. Five studies [36, 44, 51, 86, 87] reported estimates for male sex. A random-effects model was applied, and substantial between-study heterogeneity was observed (I²=90.6%, *P* < 0.05). The point estimate suggested lower odds of OP among male patients; however, the association was not statistically significant (OR = 0.40, 95% CI 0.11–1.48, *P* = 0.168). Two studies [43, 58] reported estimates for female sex. A random-effects model was applied, with moderate-to-substantial heterogeneity (I²=62.3%, *P* = 0.103). Female sex was not significantly associated with OP, and the estimate was highly imprecise (OR = 4.00, 95% CI 0.33–49.27, *P* = 0.279).

Overall, the available evidence does not support a definitive association between sex and OP among patients with COPD. The wide CIs, substantial heterogeneity, and limited number of studies, particularly in the female subgroup, indicate that these findings should be interpreted cautiously. The results are presented in Supplementary Figure S13 and Table 3.

Given the substantial heterogeneity in the sex-related analysis, a leave-one-out sensitivity analysis was performed. Exclusion of the study by Lee et al. (2017) [51] changed the pooled estimate to OR = 0.96, indicating that this study had a substantial influence on the pooled result. The study population consisted predominantly of patients with relatively mild COPD, normal or slightly elevated BMI, and limited systemic corticosteroid exposure, which may have contributed to a lower effect estimate than that observed in clinic-based populations. However, this interpretation is exploratory and does not establish male sex as a protective factor. The results are presented in Supplementary Figure S14.

Publication bias for sex as a risk factor for COPD-associated OP was assessed using a funnel plot, and Egger’s test was not statistically significant (*P* = 0.243); therefore, no statistically significant evidence of publication bias was detected in the included studies. The results are presented in Supplementary Figure S15.

### BMI

Ten studies [42, 43, 58, 79, 86] assessed the association between BMI and OP in patients with COPD; a random-effects model was used for analysis, and heterogeneity between studies was low (I² = 28.0%, *P* = 0.187). Associations were then examined across BMI categories. Five studies [42, 43, 58, 79, 86] assessed the association between being underweight and OP in patients with COPD. A random-effects model was used for analysis, and heterogeneity between studies was high (I² = 66.8%, *P* = 0.017). The findings indicated that being underweight was associated with higher odds of OP in patients with COPD (OR 3.91, 95% CI 1.36, 11.24, *P* = 0.011), a statistically significant difference. Two studies [42, 43] assessed the association between obesity and OP in patients with COPD, using a random-effects model, with low heterogeneity between studies (I² = 0.0%, *P* = 0.991). Obesity was associated with higher odds of OP in patients with COPD (OR 3.59, 95% CI 0.32–40.17, *P* = 0.300). Three studies [42, 43, 86] assessed the association between being overweight and the incidence of OP in patients with COPD, using a random-effects model; between-study heterogeneity was low (I² = 0.0%, *P* = 0.912). Overweight was associated with higher odds of OP in patients with COPD (OR 2.55, 95% CI 0.70–9.33, *P* = 0.157; see Supplementary Figure S16 and Table 3 for results).

In summary, the analysis of the association between BMI and the risk of OP in patients with COPD indicates that low body weight is significantly associated with higher odds of OP. In contrast, although the point estimates for overweight and obesity suggested higher odds, neither association reached statistical significance. These findings highlight the clinical importance of maintaining adequate nutrition and avoiding underweight status for bone health in patients with COPD, whilst the impact of excess weight on the risk of OP requires further clarification in future large-scale studies.

Given the heterogeneity in this analysis, sensitivity analyses were conducted to identify the sources of heterogeneity. After excluding the study by Kaenmuang (2023) [79], the pooled effect size showed a substantial change (OR = 4.86). As this study included a cohort not receiving anti-osteoporosis treatment, its risk estimate for low BMI (OR = 1.64) may more closely reflect the independent effect of this factor in a natural setting. In contrast, in other observational studies, the presence of ‘indication bias’ (i.e. high-risk patients being more likely to receive treatment) and the therapeutic effects of medication may have partially masked the true strength of the association between low BMI and OP, leading to diluted risk estimates. The meta-analysis reflects variation in risk estimates across different treatment-exposure contexts. The results are shown in Supplementary Figure S17.

Publication bias for BMI as a risk factor for COPD-related OP was assessed using a funnel plot, and Egger’s test was not statistically significant (*P* = 0.176); therefore, no statistically significant evidence of publication bias was detected in the included studies. The results are shown in Supplementary Figure S18.

### Corticosteroids

Eight studies [36, 42, 43, 90] assessed the association between corticosteroid exposure and OP in patients with COPD. A random-effects analysis indicated low between-study heterogeneity (I² = 24.0%, *P* = 0.238). The results showed that corticosteroid exposure was associated with higher odds of OP in patients with COPD (OR 2.85, 95% CI 1.45–5.59, *P* < 0.01). Associations were further examined according to corticosteroid dose and route of administration. Four studies [36, 42, 43, 90] assessed the association between inhaled corticosteroids (ICS) and the incidence of OP in patients with COPD. A random-effects model was used, and substantial between-study heterogeneity was observed (I² = 52.7%, *P* = 0.096). ICS exposure was associated with higher odds of OP in patients with COPD (OR 2.87, 95% CI 0.97–8.48, *P* = 0.056). Two studies [42, 43] assessed the association between steroid use (cumulative dose) < 1000 mg and the incidence of OP in patients with COPD. A random-effects model was used for analysis, and heterogeneity between studies was low (I² = 0.0%, *P* = 0.997). Steroid use (cumulative dose) < 1000 mg (prednisolone equivalent) was associated with higher odds of OP in patients with COPD (OR 1.97, 95% CI 0.42–9.34, *P* = 0.391). Two studies [42, 43] assessed the association between steroid use (cumulative dose) > 1000 mg (prednisone equivalent) and the incidence of OP in patients with COPD. A random-effects model was used for analysis, and heterogeneity between studies was low (I² = 0.0%, *P* = 0.998). Steroid use (cumulative dose) > 1000 mg (prednisone equivalent) was associated with higher odds of OP in patients with COPD (OR 7.36, 95% CI 1.70–31.98, *P* < 0.01); the results are shown in Supplementary Figure S19 and Table 3.

In summary, overall corticosteroid exposure was associated with higher odds of OP among patients with COPD (OR = 2.85, 95% CI 1.45–5.59). The association for ICS exposure did not reach statistical significance (OR = 2.87, 95% CI 0.97–8.48), and the estimate for cumulative systemic corticosteroid exposure below 1,000 mg was also not statistically significant (OR = 1.97, 95% CI 0.42–9.34). Cumulative systemic corticosteroid exposure exceeding 1,000 mg was associated with higher odds of OP (OR = 7.36, 95% CI 1.70–31.98); however, this finding was based on only two studies involving 172 participants. Therefore, the dose-stratified results should be regarded as preliminary evidence of a possible exposure-related gradient rather than confirmation of a definitive threshold or dose–response relationship.

Given the heterogeneity in this analysis, sensitivity analyses were conducted to explore potential sources of heterogeneity. After excluding the study by Graat-Verboom et al. (2009) [37], the pooled effect size changed significantly (OR = 4.47). One possible explanation is that this earlier study may reflect the clinical practices and methodological characteristics of its time, which may have differed from those of more recent studies. This suggests that, when long-term evidence is synthesised, the impact of evolving clinical guidelines and advances in research methodology on the comparability of results should be considered; see Supplementary Figure S20.

Publication bias for corticosteroid exposure as a risk factor for OP among patients with COPD was assessed using a funnel plot. Egger’s test was not statistically significant (*P* = 0.478), indicating no statistically significant evidence of publication bias in the included studies. The results are presented in Supplementary Figure S21.

#### FEV1% predicted

Three studies [47, 83, 86] assessed the association between FEV₁% predicted and OP in patients with COPD. In accordance with the prespecified analytical approach, a random-effects model was applied. No statistically significant between-study heterogeneity was observed (I²=0.0%, *P* = 0.675). The pooled analysis showed that a higher FEV₁% predicted was significantly associated with lower odds of OP (OR = 0.97, 95% CI 0.96–0.98, *P* < 0.01). The results are presented in Supplementary Figure S22 and Table 3.

Publication bias for FEV1% predicted as a risk factor for OP among patients with COPD was assessed using a funnel plot. Egger’s test was not statistically significant (*P* = 0.702); therefore, no statistically significant evidence of publication bias was detected in the included studies. The results are shown in Supplementary Figure S23.

### Age

Six studies [38, 44, 51, 83, 86, 87] assessed the association between age ≥ 65 years and OP in patients with COPD. Significant between-study heterogeneity was observed (I² = 88.7%, *P* < 0.05); therefore, a random-effects model was applied. The pooled point estimate suggested higher odds of OP among patients aged ≥ 65 years, but this did not reach statistical significance (OR = 1.04, 95% CI 0.99–1.09, *P* = 0.121); see Supplementary Figure S24 and Table 3.

Given the heterogeneity in this analysis, a sensitivity analysis was conducted to investigate its source. After excluding the study by Bitar et al. [83], the pooled effect size changed slightly (OR = 1.05). The analysis suggests that this discrepancy may reflect the inclusion of only male patients with COPD in the study by Bitar et al., which excluded postmenopausal women, a high-risk population. This may have produced a lower risk estimate for ‘age ≥ 65 years’ than in studies that included both sexes, thereby constituting a significant source of heterogeneity in this meta-analysis; see Supplementary Figure S25.

A funnel plot was used to assess publication bias, and Egger’s test was not statistically significant (*P* = 0.897), providing no statistically significant evidence of publication bias in the included studies. The results are presented in Supplementary Figure S26.

**Table 3:** Summary of Risk factors for OP in COPD

Subgroup category	Specific classification	Number of studies within this subgroup	Total sample size(*n*)	Heterogeneity I2	Effect size OR(95%CI)	*P* Value
Sex	Male	5	1921	90.6%	0.40(0.11,1.48)	0.168
	Female	2	187	62.3%	4.00(0.33,49.27)	0.279
BMI	BMI Overweight	3	263	0.0%	2.55(0.70,9.33)	0.157
	BMI Obese	2	172	0.0%	3.59(0.32,40.17)	0.300
	BMI Underweight	5	456	66.8%	3.91(1.36,11.24)	0.011
Corticosteroids	Total	8	813	24.0%	2.85(1.45,5.59)	*P*<0.01
	Corticosteroids Inhaled	4	813	52.7%	2.87(0.97,8.48)	0.056
	Steroid Use (Cumulative Dose) <1000 mg	2	172	0.0%	1.97(0.42,9.34)	0.391
	Steroid Use (Cumulative Dose) > 1000 mg	2	172	0.0%	7.36(1.70,31.98)	*P*<0.01
FEV1% predicted	Total	3	671	0.0%	0.97(0.96,0.98)	*P*<0.01
Age ≥ 65	Total	6	2002	88.7%	1.04(0.99,1.09)	0.121

## Discussion

### Key findings

This systematic review and meta-analysis included 87 studies involving 1,453,879 patients with COPD. The pooled prevalence of OP was 33.8%, indicating a substantial disease burden. However, between-study heterogeneity was extremely high (I²=99.7%). Therefore, the pooled prevalence should be interpreted as an average estimate across studies with diverse clinical and methodological characteristics rather than as a universally applicable prevalence value.

Among the clinical factors examined, underweight was significantly associated with higher odds of OP (OR = 3.91, 95% CI 1.36–11.24). Corticosteroid exposure was also associated with higher odds of OP (OR = 2.85, 95% CI 1.45–5.59). In the dose-stratified analysis, cumulative systemic corticosteroid exposure exceeding 1,000 mg prednisone-equivalent was associated with higher odds of OP (OR = 7.36, 95% CI 1.70–31.98). However, this estimate was based on only two studies involving 172 participants and should therefore be regarded as preliminary evidence suggesting a possible exposure-related gradient rather than as confirmation of a definitive threshold or dose–response relationship.

Higher FEV₁% predicted was significantly associated with lower odds of OP (OR = 0.97, 95% CI 0.96–0.98). In contrast, no statistically significant associations were identified for sex, age ≥ 65 years, overweight status, or obesity. These non-significant findings should not be interpreted as evidence of protective effects. Overall, the results suggest that underweight, corticosteroid exposure, and impaired lung function may be clinically relevant factors associated with OP among patients with COPD, although residual confounding, inconsistent covariate adjustment, and limited evidence for several analyses should be considered.

#### Comparison with other similar studies

The pooled OP prevalence of 33.8% observed in the present study is broadly consistent with, although slightly lower than, the approximately 38% reported by Chen et al. [15]. The difference in point estimates may reflect the substantial expansion of the evidence base, differences in the composition and characteristics of the included studies, and the inclusion of several large database-based populations in the present review. Nevertheless, both reviews demonstrate considerable between-study variability, reinforcing that a single pooled prevalence estimate should be interpreted cautiously across clinically and methodologically diverse settings.

The substantial heterogeneity observed in the present analysis is also consistent with the methodological variability highlighted in previous evidence syntheses. Penedones et al. [16] identified considerable differences across studies in population characteristics, diagnostic approaches, and outcome definitions. Building on these observations, the present study quantitatively explored several study-level characteristics using subgroup analyses and univariable meta-regression. Methodological quality score was significantly associated with variation in prevalence estimates, whereas publication year showed a possible trend towards an association. In contrast, study design, sample size, continent, mean participant age, and BMD assessment method were not significantly associated with variation in prevalence estimates. Although the pooled prevalence was numerically higher in studies using DXA than in those using non-DXA methods, this difference was not supported by the meta-regression analysis. These findings suggest that methodological differences may partly contribute to the variability observed across studies, although substantial residual heterogeneity remains unexplained.

These findings should, however, be interpreted in the context of the limitations of study-level meta-regression. The present analyses were univariable and based on aggregate study-level characteristics and therefore cannot establish independent or causal determinants of heterogeneity. Important clinical and contextual factors, including COPD severity, sex distribution, corticosteroid exposure, smoking history, physical activity, comorbidities, and healthcare-related characteristics, could not be reliably incorporated because of incomplete or inconsistent reporting across the included studies. Thus, the present findings extend previous descriptions of heterogeneity but do not fully resolve its underlying causes.

With regard to clinically relevant associated factors, the present findings are broadly consistent with previous evidence identifying low body weight as an important marker of skeletal vulnerability in COPD. Underweight was significantly associated with higher odds of OP, whereas overweight and obesity were not significantly associated with OP in the pooled adjusted analyses. Importantly, these non-significant findings should not be interpreted as evidence of protective effects because the corresponding analyses were based on relatively few studies and produced imprecise estimates.

The present study also provides a more detailed assessment of corticosteroid exposure than a general pooled analysis alone. Overall corticosteroid exposure was associated with higher odds of OP, whereas the association for ICS exposure did not reach statistical significance. Huang et al. [17] specifically examined long-term ICS exposure in relation to OP and fracture outcomes and similarly highlighted uncertainty regarding the skeletal effects of ICS. In the present study, cumulative systemic corticosteroid exposure exceeding 1,000 mg prednisone-equivalent was associated with higher odds of OP, whereas exposure below 1,000 mg was not statistically significant. However, the dose-stratified analysis was based on only two studies involving 172 participants and yielded wide CIs. Therefore, these findings should be regarded as preliminary evidence of a possible exposure-related gradient rather than confirmation of a definitive threshold or dose–response relationship.

In addition, higher FEV₁% predicted was significantly associated with lower odds of OP, suggesting that poorer pulmonary function may be linked to greater skeletal vulnerability. However, this association may also be influenced by COPD severity, physical inactivity, nutritional status, systemic inflammation, and corticosteroid exposure. Because the pooled risk-factor analyses were based exclusively on adjusted ORs, the present study avoided directly combining adjusted and unadjusted estimates; nevertheless, differences in the covariates included in the original multivariable models mean that residual confounding cannot be excluded.

Overall, the principal added value of the present study lies not in reconfirming the well-established coexistence of COPD and OP, but in providing a broader and more up-to-date quantitative synthesis of the available evidence, extending the quantitative exploration of potential methodological contributors to heterogeneity, and providing a more granular assessment of selected clinically relevant factors. These findings reinforce the substantial burden of OP among patients with COPD while also highlighting the considerable uncertainty that remains because of methodological heterogeneity, inconsistent reporting, and limited evidence for several risk-factor analyses. Future studies using standardised diagnostic criteria and more detailed reporting of disease severity, medication exposure, clinical characteristics, and potential confounders are needed to improve the comparability of evidence and support more reliable risk stratification.

### In-depth interpretation of findings and pathophysiological mechanisms

Our findings quantify the burden of OP in COPD and provide insights into the mechanisms underlying the “lung–bone axis.” The 33.8% prevalence may be partly related to chronic systemic inflammation, in which pro-inflammatory cytokines (TNF-α, IL-1β, and IL-6) disrupt skeletal homeostasis by activating osteoclasts through the RANKL/RANK/OPG pathway and suppressing osteoblast function through the Wnt/β-catenin signalling cascade, thereby contributing to bone loss [5, 6, 106–113]. This inflammatory milieu may interact with therapeutic and nutritional determinants. Cumulative systemic corticosteroid dose emerged as a potentially modifiable factor, and the available data suggested a possible threshold-related pattern: cumulative doses exceeding 1,000 mg were associated with higher odds of OP. This is consistent with corticosteroid-induced osteoblast apoptosis, inhibition of bone formation, and disruption of calcium–phosphorus metabolism [114–116]. The association with low body weight (BMI < 18.5 kg/m²) is consistent with the “pulmonary cachexia–sarcopenia–osteoporosis” syndrome, in which lean tissue depletion reduces mechanical loading and endocrine protection while interacting with nutritional deficiencies to accelerate skeletal deterioration [117–119].

These mechanisms may help explain the patterns observed in our stratified analyses. The inverse association between FEV₁% predicted and OP suggests that worsening lung function may reflect systemic hypoxia, oxidative stress, and physical inactivity, each of which can impair bone metabolism [120–122]. The higher odds observed in underweight individuals contrasted with the non-significant findings for overweight and obesity. This pattern may partly reflect mechanical loading and peripheral oestrogen conversion associated with adipose tissue, potentially offsetting detrimental effects of adiposity-related inflammation [123–126]. Similarly, the non-significant association for age ≥ 65 years does not exclude a contribution from biological ageing and may partly reflect confounding by preventive therapies, such as calcium, vitamin D, and anti-resorptive treatment, more commonly prescribed to older adults. Finally, the possible dose-related association with corticosteroid exposure supports careful corticosteroid stewardship, as higher cumulative doses may cause greater osteocyte-network damage, whereas lower doses were not clearly associated with higher odds of OP [127, 128]. ICS exposure showed a weaker trend towards higher odds of OP, possibly because of lower systemic bioavailability, although long-term high-dose use still warrants vigilance [129]. Collectively, FEV₁% predicted may serve as a useful integrative marker of skeletal risk, reflecting combined therapeutic, nutritional, and systemic disease burdens in patients with COPD [130–132].

### Potential confounding and interpretation of the observed associations

Because all included primary studies were observational, the associations identified in this meta-analysis remain susceptible to residual and unmeasured confounding, even though the pooled risk-factor estimates were based exclusively on adjusted ORs. Age, sex, and menopausal status are particularly important because they are strongly associated with bone loss, and their distributions may also vary according to COPD severity, treatment patterns, and study setting. Inadequate adjustment for these factors could exaggerate or attenuate the observed associations between COPD-related characteristics and OP.

Smoking is another important potential confounder. Greater cumulative smoking exposure is associated with more severe airflow limitation and may also adversely affect bone metabolism. Therefore, failure to account adequately for smoking intensity and duration may partly inflate the association between reduced FEV₁% predicted and OP. Similarly, physical inactivity may be more common in patients with severe COPD and may independently contribute to muscle loss, reduced mechanical loading, and lower BMD. However, physical inactivity may also lie on the causal pathway between COPD severity and OP; consequently, adjusting for it may either reduce confounding or remove part of the true disease-related effect, depending on the causal question.

Corticosteroid exposure is closely related to disease severity and may introduce confounding by indication. Patients with more severe COPD or frequent exacerbations are more likely to receive systemic corticosteroids and to have other characteristics associated with OP. Thus, the observed association between corticosteroid exposure and OP may partly reflect underlying disease severity, whereas adjustment for COPD severity may attenuate the estimated association. Conversely, incomplete measurement of cumulative dose, duration, and route of administration may lead to residual confounding and exposure misclassification.

Nutritional status, BMI, muscle mass, vitamin D status, and systemic inflammation are also interrelated. Low BMI may represent both an independent marker of skeletal vulnerability and a consequence of advanced COPD-related cachexia. Therefore, the association between low BMI and OP may partly reflect disease severity, reduced physical activity, and nutritional deficiency. Comorbidities such as cardiovascular disease, diabetes, renal dysfunction, and endocrine disorders, together with medications affecting bone metabolism, may further modify the observed associations.

Finally, preventive interventions, including calcium, vitamin D, anti-resorptive treatment, pulmonary rehabilitation, and better access to healthcare, may attenuate associations in populations receiving more intensive care. In contrast, limited access to screening and treatment may result in underdiagnosis of OP in resource-constrained settings. Accordingly, the pooled adjusted ORs should be interpreted as associations rather than causal effects. Differences in the covariates included in the original multivariable models may also have contributed to the variation in effect estimates across studies.

## Strengths and limitations

### Strengths

This study has several strengths. First, it represents one of the largest and most comprehensive meta-analyses to date examining the prevalence of OP and its associated factors among patients with COPD, including 87 studies and more than 1.45 million participants. The broad database search strategy and updated evidence base enabled a comprehensive synthesis of the available epidemiological literature.

Second, the study applied explicit eligibility criteria requiring objectively documented COPD and OP diagnoses and used independent study selection, data extraction, and methodological quality assessment. The NOS and AHRQ assessment tools were applied according to study design, allowing the methodological quality of cohort, case-control, and cross-sectional studies to be evaluated using appropriate frameworks.

Third, a random-effects model was specified a priori for all quantitative syntheses in view of the anticipated clinical and methodological diversity. Potential sources of heterogeneity were explored using subgroup analyses, leave-one-out sensitivity analyses, and univariable meta-regression examining several study-level characteristics, including geographical region, study design, mean age, BMD assessment method, publication year, sample size, and methodological quality.

Fourth, all pooled analyses of factors associated with OP were based exclusively on adjusted ORs, thereby avoiding the direct combination of adjusted and unadjusted estimates. The analyses also examined selected clinically relevant factors separately, including BMI categories, corticosteroid route and cumulative exposure, FEV₁% predicted, age, and sex.

Finally, the exploratory dose-stratified analysis of systemic corticosteroid exposure provides preliminary quantitative evidence concerning a possible exposure-related gradient. Although this finding requires confirmation because of the limited number of contributing studies, it identifies an important area for future clinical and epidemiological research.

### Limitations

The extremely high residual heterogeneity indicates that the pooled prevalence should be interpreted as an average prevalence estimate across clinically and methodologically diverse settings rather than as a universally applicable prevalence value. Although subgroup analyses and study-level meta-regression were conducted to explore several potential sources of heterogeneity, including geographical region, study design, mean participant age, BMD assessment method, publication year, sample size, and methodological quality, these analyses could explain only part of the observed variability.

Although we explored BMD assessment methods by comparing DXA with non-DXA methods, more detailed diagnostic characteristics could not be quantitatively evaluated. The included studies differed in the manufacturers of the DXA equipment used, the diagnostic thresholds applied, and the skeletal measurement sites assessed, including the lumbar spine, femoral neck, and total hip. In several studies, OP was defined according to the lowest T-score across multiple skeletal sites, whereas site-specific prevalence estimates were not separately reported. Similarly, COPD severity was reported using heterogeneous measures, including GOLD stages, FEV₁% predicted, symptom scores, exacerbation history, and oxygen use. Consequently, specific DXA device characteristics, skeletal measurement sites, and GOLD stages could not be reliably incorporated into subgroup analyses or meta-regression. The contribution of these characteristics to the substantial residual heterogeneity therefore remains unresolved.

Mean age was analysed as a continuous study-level covariate to supplement the broad age subgroup analysis. However, study-level mean age cannot capture within-study age distributions, nonlinear age-related effects, or interactions between age and other individual characteristics. Moreover, potentially important clinical factors, including COPD severity, comorbidities, medication exposure, physical activity, smoking history, and preventive treatment for OP, were inconsistently reported or defined across the included studies and therefore could not be reliably evaluated through subgroup analyses or meta-regression.

Contextual and sociodemographic determinants, such as socioeconomic status, occupation, household conditions, environmental exposures, healthcare access, insurance status, and health-seeking behaviour, were rarely or inconsistently reported. The potential contribution of these determinants to between-study heterogeneity therefore remains unresolved. These limitations highlight the need for future studies to adopt standardised reporting frameworks and provide more detailed individual-level clinical and sociodemographic data.

Although our inclusion criteria permitted BMD-based diagnoses and clearly specified clinical or administrative case definitions, the requirement for documented and verifiable diagnostic criteria may still limit the generalisability of our findings. Access to spirometry for COPD diagnosis, BMD assessment for OP, specialist evaluation, and standardised diagnostic coding remains uneven worldwide, particularly in low- and middle-income countries. Consequently, studies from well-resourced healthcare systems may be overrepresented, whereas the burden of COPD-related OP in resource-constrained settings may be underestimated because of limited diagnostic access and potential underdiagnosis. This diagnostic access bias should be considered when extrapolating the pooled estimates to regions with limited healthcare and diagnostic infrastructure.

Two additional sources of bias warrant acknowledgement. First, our restriction to English-language publications may have introduced language bias. Given that a significant proportion of research from low- and middle-income countries (LMICs), which bear a disproportionate burden of COPD, is published in regional languages or non-English journals, this criterion likely led to the underrepresentation of studies from these high-prevalence regions. This may limit the applicability of our findings to LMIC settings where diagnostic resources and disease patterns may differ from high-income countries. Second, the exclusion of grey literature (e.g., unpublished theses, conference abstracts, governmental reports) may have contributed to publication bias, as studies with null or negative findings are less likely to be formally published. This bias could lead to an overestimation of the true prevalence of COPD-related osteoporosis because smaller studies with non-significant results are systematically underrepresented in our analysis. These limitations underscore the need for future reviews to incorporate multilingual search strategies and grey literature sources to enhance the global representativeness of pooled estimates.

Although all pooled estimates were adjusted ORs, the covariates included in the original multivariable models were not necessarily identical across studies and were not consistently reported in all publications. Therefore, differences in covariate adjustment and residual or unmeasured confounding may still have influenced the pooled associations.

### Implications for clinical practice and future research

#### Clinical practice

Our findings highlight the importance of bone health assessment and management in patients with COPD. Particular attention may be warranted for patients who are underweight, have substantial systemic corticosteroid exposure, or have more severe airflow limitation, as these factors were associated with higher odds of OP in the present meta-analysis. Bone mineral density assessment, including DXA where clinically appropriate, may be considered part of an individualised risk assessment strategy in these higher-risk patients. Management should also emphasise adequate nutritional support, appropriate physical activity to preserve musculoskeletal health, and judicious use of corticosteroids to minimise potentially avoidable adverse effects on bone health.

#### Future research

High-quality, prospective cohort studies are urgently needed. Future investigations should adopt standardised diagnostic protocols for OP and systematically collect data on key confounders (e.g., medication history, biomarkers of bone turnover and inflammation, and body composition). Research should also explore the longitudinal relationship between indicators of COPD progression, such as the frequency of acute exacerbations and functional decline, and bone loss, while evaluating the efficacy of targeted interventions, such as resistance-based pulmonary rehabilitation, with respect to bone density outcomes.

## Conclusion

Overall, OP affects approximately one-third of patients with COPD across the included studies. Underweight, corticosteroid exposure, and lower FEV₁% predicted were associated with OP and may represent clinically relevant factors for risk assessment. The dose-stratified analysis suggested that greater cumulative systemic corticosteroid exposure may be associated with higher odds of OP; however, the limited number of studies does not permit confirmation of a definitive dose threshold or dose–response relationship.

Given the extremely high residual heterogeneity, variation in covariate adjustment, and potential residual or unmeasured confounding, the pooled estimates should be interpreted cautiously. Future prospective studies that use standardised COPD and OP diagnostic criteria, apply consistent methods for measuring corticosteroid exposure, and comprehensively report clinical and sociodemographic characteristics are needed to improve risk estimation and inform targeted bone-health management in patients with COPD.

## Supplementary Information

Supplementary Material 1.

Supplementary Material 2.

## Data Availability

All data analyzed during this study are included in the published articles retrieved from public databases. The datasets generated and/or analyzed during the current study are available from the corresponding author on reasonable request.

## Abbreviations

AHRQ: Agency for Healthcare Research and Quality
BMD: Bone mineral density
BMI: Body mass index
CI: Confidence interval
COPD: Chronic obstructive pulmonary disease
DXA: Dual-energy X-ray absorptiometry
FEV₁: Forced expiratory volume in 1 s
GOLD: Global Initiative for Chronic Obstructive Lung Disease
ICS: Inhaled corticosteroids
LMICs: Low- and middle-income countries
NOS: Newcastle–Ottawa Scale
OP: Osteoporosis
OR: Odds ratio
PROSPERO: International Prospective Register of Systematic Reviews
QCT: Quantitative computed tomography
QUS: Quantitative ultrasound
