# Supplementary material for: Global prevalence and risk factors of osteoporosis in patients with chronic obstructive pulmonary disease: a systematic review and meta-analysis
Source: BMC Public Health. 2026 Aug 19;26:3375. doi: 10.1186/s12889-026-28811-1 (PMC13760868; doi:10.1186/s12889-026-28811-1)
Supplement: Supplementary file 2 [file 12889_2026_28811_MOESM2_ESM.docx]

**SUPPLEMENTARY MATERIAL**

**Table of Contents**

**Methods 1——Search Strategy	1**

**Table S1: Demographic characteristics of included studies.	4**

**Table S2: Newcastle-Ottawa Quality Assessment for Cohort and Case-Control Studies	20**

**Table S3: Cross-Sectional/Prevalence Study Quality	23**

**Figure S1: Forest Plot:The overall prevalence of COPD with OP and the corresponding subgroup analysis by continent are presented.	28**

**Figure S2: Forest Plot:The prevalence of COPD with OP stratified by Study design is presented.	29**

**Figure S3: Forest Plot:The prevalence of COPD with OP stratified by BMI is presented.	30**

**Figure S4: Forest Plot:The prevalence of COPD with OP stratified by Age is presented.	31**

**Figure S5:** **Forest Plot:The prevalence of COPD with OP stratified by BMD assessment method is presented.	32**

**Figure S6: Scatter plot with regression line of effect size (ES) by number.	33**

**Figure S7:Scatter plot with regression line of effect size (ES) by continent.	33**

**Figure S8:Scatter plot with regression line of effect size (ES) by year.	34**

**Figure S9:Scatter plot with regression line of effect size (ES) by Literature quality score.	34**

**Figure S10:Scatter plot with regression line of effect size (ES) by Study design.	35**

**Figure S11:Scatter plot with regression line of effect size (ES) by Mean participant age.	35**

**Figure S12:Scatter plot with regression line of effect size (ES) by BMD assessment method.	36**

**Figure S13: Forest Plot:Risk factors for COPD with OP stratified by Sex	36**

**Figure S14: Sensitivity analysis:Risk factors for COPD with OP stratified by Sex	37**

**Figure S15: Funnel plot:Risk factors for COPD with OP stratified by Sex	37**

**Figure S16: Forest Plot:Risk factors for COPD with OP stratified by BMI	38**

**Figure S17: Sensitivity analysis:Risk factors for COPD with OP stratified by BMI	38**

**Figure S18: Funnel plot:Risk factors for COPD with OP stratified by BMI	39**

**Figure S19: Forest Plot:Risk factors for COPD with OP stratified by Corticosteroids	39**

**Figure S20: Sensitivity analysis:Risk factors for COPD with OP stratified by Corticosteroids	40**

**Figure S21: Funnel plot:Risk factors for COPD with OP stratified by Corticosteroids	40**

**Figure S22: Forest Plot:Risk factors for COPD with OP stratified by FEV1% predicted	41**

**Figure S23: Funnel plot:Risk factors for COPD with OP stratified by FEV1% predicted	41**

**Figure S24: Forest Plot:Risk factors for COPD with OP stratified by Age	42**

**Figure S25: Sensitivity analysis:Risk factors for COPD with OP stratified by Age	42**

**Figure S26: Funnel plot:Risk factors for COPD with OP stratified by Age	43**

**Supplementary Methods 1——Search Strategy**

PUBMED

Search: (((("Pulmonary Disease, Chronic Obstructive"[Mesh]) AND (Pulmonary Disease, Chronic Obstructive[Title/Abstract])) OR ((((((((((Chronic Obstructive Pulmonary Diseases[Title/Abstract]) OR (COPD[Title/Abstract])) OR (Chronic Obstructive Lung Disease[Title/Abstract])) OR (Chronic Obstructive Pulmonary Disease[Title/Abstract])) OR (COAD[Title/Abstract])) OR (Chronic Obstructive Airway Disease[Title/Abstract])) OR (Airflow Obstruction, Chronic[Title/Abstract])) OR (Airflow Obstructions, Chronic[Title/Abstract])) OR (Chronic Airflow Obstructions[Title/Abstract])) OR (Chronic Airflow Obstruction[Title/Abstract]))) AND ((("Osteoporosis"[Mesh]) AND (Osteoporosis[Title/Abstract])) OR ((((((((((((((((((((((Osteoporoses[Title/Abstract]) ) OR (Osteoporosis, Age-Related[Title/Abstract])) OR (Osteoporosis, Age Related[Title/Abstract])) OR (Age-Related Osteoporosis[Title/Abstract])) OR (Age-Related Osteoporoses[Title/Abstract])) OR (Age Related Osteoporosis[Title/Abstract])) OR (Osteoporoses, Age-Related[Title/Abstract])) OR (Bone Loss, Age-Related[Title/Abstract])) OR (Age-Related Bone Loss[Title/Abstract])) OR (Age-Related Bone Losses[Title/Abstract])) OR (Bone Loss, Age Related[Title/Abstract])) OR (Bone Losses, Age-Related[Title/Abstract])) OR (Osteoporosis, Senile[Title/Abstract])) OR (Osteoporoses, Senile[Title/Abstract])) OR (Senile Osteoporoses[Title/Abstract])) OR (Senile Osteoporosis[Title/Abstract])) OR (Osteoporosis, Involutional[Title/Abstract])) OR (Osteoporosis, Post-Traumatic[Title/Abstract])) OR (Osteoporosis, Post Traumatic[Title/Abstract])) OR (Post-Traumatic Osteoporoses[Title/Abstract])) OR (Post-Traumatic Osteoporosis[Title/Abstract])))) AND (prevalence[Title/Abstract] OR risk factors[Title/Abstract] OR influence factor[Title/Abstract])

Scopus

（"chronic obstructive pulmonary disease" OR "Chronic Obstructive Pulmonary Diseases" OR "COPD" OR "Chronic Obstructive Lung Disease" OR "Chronic Obstructive Pulmonary Disease" OR "COAD" OR "Chronic Obstructive Airway Disease" OR "Airflow Obstruction, Chronic" OR "Airflow Obstructions, Chronic" OR "Chronic Airflow Obstructions" OR "Chronic Airflow Obstruction"） AND ("Osteoporosis" OR "Osteoporoses" OR "Osteoporosis, Age-Related" OR "Osteoporosis, Age Related" OR "Age-Related Osteoporosis" OR "Age-Related Osteoporoses" OR "Age Related Osteoporosis" OR "Osteoporoses, Age-Related" OR "Bone Loss, Age-Related" OR "Age-Related Bone Loss" OR "Age-Related Bone Losses" OR "Bone Loss, Age Related" OR "Bone Losses, Age-Related" OR "Osteoporosis, Senile" OR "Osteoporoses, Senile" OR "Senile Osteoporoses" OR "Senile Osteoporosis" OR "Osteoporosis, Involutional" OR "Osteoporosis, Post-Traumatic" OR "Osteoporosis, Post Traumatic" OR "Post-Traumatic Osteoporoses" OR "Post-Traumatic Osteoporosis") AND ("prevalence" OR "risk factors" OR "influence factor")

EMBASE

('chronic obstructive lung disease'/exp OR 'chronic airflow obstruction':ab,ti OR 'chronic airway obstruction':ab,ti OR 'chronic obstructive bronchopulmonary disease':ab,ti OR 'chronic obstructive lung disorder':ab,ti OR 'chronic obstructive pulmonary disease':ab,ti OR 'chronic obstructive pulmonary disorder':ab,ti OR 'chronic obstructive respiratory disease':ab,ti OR 'chronic pulmonary obstructive disease':ab,ti OR 'chronic pulmonary obstructive disorder':ab,ti OR 'copd':ab,ti OR 'lung chronic obstructive disease':ab,ti OR 'lung disease, chronic obstructive':ab,ti OR 'obstructive chronic lung disease':ab,ti OR 'obstructive chronic pulmonary disease':ab,ti OR 'obstructive lung disease, chronic':ab,ti OR 'pulmonary disease, chronic obstructive':ab,ti OR 'pulmonary disorder, chronic obstructive':ab,ti OR 'chronic obstructive lung disease':ab,ti) AND ('osteoporosis'/exp OR 'Osteoporoses':ab,ti OR 'Osteoporosis, Age-Related':ab,ti OR 'Osteoporosis, Age Related':ab,ti OR 'Age-Related Osteoporosis':ab,ti OR 'Age-Related Osteoporoses':ab,ti OR 'Age Related Osteoporosis':ab,ti OR 'Osteoporoses, Age-Related':ab,ti OR 'Bone Loss, Age-Related':ab,ti OR 'Age-Related Bone Loss':ab,ti OR 'Age-Related Bone Losses':ab,ti OR 'Bone Loss, Age Related':ab,ti OR 'Bone Losses, Age-Related':ab,ti OR 'Osteoporosis, Senile':ab,ti OR 'Osteoporoses, Senile':ab,ti OR 'Senile Osteoporoses':ab,ti OR 'Senile Osteoporosis':ab,ti OR 'Osteoporosis, Involutional':ab,ti OR 'Osteoporosis, Post-Traumatic':ab,ti OR 'Osteoporosis, Post Traumatic':ab,ti OR 'Post-Traumatic Osteoporoses':ab,ti OR 'Post-Traumatic Osteoporosis':ab,ti) AND ('prevalence':ab,ti OR 'risk factors':ab,ti OR 'influence factor':ab,ti)

CINAHL

[((SU (prevalence OR risk factors OR influence factor)) OR (TI (prevalence OR risk factors OR influence factor)) OR (AB (prevalence OR risk factors OR influence factor))) AND ((( ((SU ("chronic obstructive pulmonary disease") OR TI (“Chronic Obstructive Pulmonary Diseases" OR "COPD" OR "Chronic Obstructive Lung Disease" OR "Chronic Obstructive Pulmonary Disease" OR "COAD" OR "Chronic Obstructive Airway Disease" OR "Airflow Obstruction, Chronic" OR "Airflow Obstructions, Chronic" OR "Chronic Airflow Obstructions" OR "Chronic Airflow Obstruction") OR AB (“Chronic Obstructive Pulmonary Diseases" OR "COPD" OR "Chronic Obstructive Lung Disease" OR "Chronic Obstructive Pulmonary Disease" OR "COAD" OR "Chronic Obstructive Airway Disease" OR "Airflow Obstruction, Chronic" OR "Airflow Obstructions, Chronic" OR "Chronic Airflow Obstructions" OR "Chronic Airflow Obstruction")) AND (SU ("osteoporosis") OR TI (“Osteoporoses" OR "Osteoporosis, Age-Related" OR "Osteoporosis, Age Related" OR "Age-Related Osteoporosis" OR "Age-Related Osteoporoses" OR "Age Related Osteoporosis" OR "Osteoporoses, Age-Related" OR "Bone Loss, Age-Related" OR "Age-Related Bone Loss" OR "Age-Related Bone Losses" OR "Bone Loss, Age Related" OR "Bone Losses, Age-Related" OR "Osteoporosis, Senile" OR "Osteoporoses, Senile" OR "Senile Osteoporoses" OR "Senile Osteoporosis" OR "Osteoporosis, Involutional" OR "Osteoporosis, Post-Traumatic" OR "Osteoporosis, Post Traumatic" OR "Post-Traumatic Osteoporoses" OR "Post-Traumatic Osteoporosis") OR AB (““Osteoporoses" OR "Osteoporosis, Age-Related" OR "Osteoporosis, Age Related" OR "Age-Related Osteoporosis" OR "Age-Related Osteoporoses" OR "Age Related Osteoporosis" OR "Osteoporoses, Age-Related" OR "Bone Loss, Age-Related" OR "Age-Related Bone Loss" OR "Age-Related Bone Losses" OR "Bone Loss, Age Related" OR "Bone Losses, Age-Related" OR "Osteoporosis, Senile" OR "Osteoporoses, Senile" OR "Senile Osteoporoses" OR "Senile Osteoporosis" OR "Osteoporosis, Involutional" OR "Osteoporosis, Post-Traumatic" OR "Osteoporosis, Post Traumatic" OR "Post-Traumatic Osteoporoses" OR "Post-Traumatic Osteoporosis")))))](http://research--ebsco--com--https.ebscohost.shd1rmyy.lwnote.com:50001/search/results?combinedSearchQueryId=sq:99816908-5329-45d7-b4fd-e199d742ef52&db=ccm&expanders=concept&limiters=None&searchMode=boolean&sort=relevance&sqId=sq:99816908-5329-45d7-b4fd-e199d742ef52&userDirectAction=true)

Cochrane Library

(MeSH descriptor: [Pulmonary Disease, Chronic Obstructive] explode all trees OR (Chronic Obstructive Pulmonary Diseases OR COPD OR Chronic Obstructive Lung Disease OR Chronic Obstructive Pulmonary Disease OR COAD OR Chronic Obstructive Airway Disease OR Airflow Obstruction, Chronic OR Airflow Obstructions, Chronic OR Chronic Airflow Obstructions OR Chronic Airflow Obstruction):ti,ab,kw ) AND (MeSH descriptor: [Osteoporosis] explode all trees OR (Osteoporoses OR Osteoporosis, Age-Related OR Osteoporosis, Age Related OR Age-Related Osteoporosis OR Age-Related Osteoporoses OR Age Related Osteoporosis OR Osteoporoses, Age-Related OR Bone Loss, Age-Related OR Age-Related Bone Loss OR Age-Related Bone Losses OR Bone Loss, Age Related OR Bone Losses, Age-Related OR Osteoporosis, Senile OR Osteoporoses, Senile OR Senile Osteoporoses OR Senile Osteoporosis OR Osteoporosis, Involutional OR Osteoporosis, Post-Traumatic OR Osteoporosis, Post Traumatic OR Post-Traumatic Osteoporoses OR Post-Traumatic Osteoporosis):ti,ab,kw) AND (prevalence OR risk factors OR influence factor):ti,ab,kw

# Supplementary Table S1: Demographic characteristics of included studies.

| First Author (Year) | Continent | COPD Sample size n (M/F) | OP Sample size n | Age | FEV1 (% predicted) | BMI（kg/m²） | Device | Location | Criteria | Type of English literature |
| --- | --- | --- | --- | --- | --- | --- | --- | --- | --- | --- |
| Abbasi (2016) [18] | Asia | 89 (89/0) | 47 | 69 (6) | NA | 22.2 (4.2) | DXA | Lumbar spine  Femoral neck | WHO | cross-sectional study |
| Arenas-de Larriva(2010)[19] | Europe | 64 | 36 | NA | NA | NA | DXA | Lumbar spine  Femoral neck | WHO | cross-sectional study |
| Bhattacharyya(2011)[20] | Asia | 37 (35/2) | 8 | 65.3 (9.6) | NA | NA | Broadband  ultrasound bone  densitometer | Calcaneus | WHO | cross-sectional study |
| Bolton(2004)[21] | Europe | 81 (43/38) | 26 | 65.8 | 44% | 23.9 | DXA | Lumbar spine  Hip | WHO | cross-sectional study |
| Bolton(2008)[22] | Europe | 58 (31/27) | 14 | 66.8 (8.7) | 54.17 ± 20.18% | 25.6 (4.5) | DXA | Lumbar spine  Hip | WHO | cross-sectional study |
| Bolton(2009)[23] | Europe | 70 (40/30) | 19 | 61.6 (9.2) | 47% | 24.4 (5.0) | DXA | Lumbar spine  Hip | WHO | cross-sectional study |
| Bon (2010)[24] | North America | 40 (16/24) | 17 | 59 (6) | 23.5 ± 8.3% | NA | DXA | Lumbar spine  Hip | WHO | cross-sectional study |
| Chubachi(2014)[25] | Asia | 210 (188/22) | 38 | 72.8 (8.3) | 65.4 ± 20.4% | 22.8 (3.3) | DXA | Lumbar spine  Hip | WHO | cross-sectional study |
| Coin(2010)[26] | Europe | 41 (41/0) | 12 | 75.7 (5.3) | 53.1 ± 17.6% | 25.4 (3.5) | DXA | Lumbar spine  Hip | WHO | cross-sectional study |
| Dimai(2001)[27] | North America | 71 (71/0) | 20 | 64.5 (0.9) | NA | 25.8 (0.4) | QCT | Distal forearm | WHO | cross-sectional study |
| Dubois(2002)[28] | Europe | 86 (86/0) | 27 | 64.6 | NA | 24.9 | DXA | Lumbar spine  Hip  Femoral neck | WHO | cross-sectional study |
| Duckers(2011)[29] | Europe | 30 (30/0) | 5 | 66 (8.5) | 63.7 ± 17.9% | 26.4 (5.1) | DXA | Lumbar spine  Hip | WHO | cross-sectional study |
| Xu（2020）[30] | Asia | 133(0/133) | 50 | 61.40 ± 6.42 | 64.90% ± 7.79% | 21.88 ± 3.30 | DXA | left femoral neck | WHO | cross-sectional study |
| Fouda(2017)[31] | Asia | 52 (49/3) | 19 | 63.9 | 50.0 ± 13.1% | 27.4 | DXA | Lumbar spine  Femoral neck | WHO | cross-sectional study |
| Fountoulis (2012)[32] | Europe | 46 (46/0) | 9 | 69.1 (8.7) | NA | NA | DXA | Whole body  Lumbar spine  Hip | WHO | cross-sectional study |
| Fountoulis (2016）[33] | Europe | 37 (37/0) | 9 | 67.8 (7.5) | NA | 27.2 (4.6) | DXA | Lumbar spine | WHO | cross-sectional study |
| Franco(2009)[34] | South America | 49 (21/28) | 25 | 65.4 (9.2) | NA | 25.8 (5.3) | DXA | Lumbar spine  Femoral neck  Total femur | International Society  for Clinical  Densitometry | cross-sectional study |
| Gonçalves(2017)[35] | South America | 61 (45/16) | 22 | 65.1 (6.1) | NA | 25.9 (5.2) | DXA | Lumbar spine Femoral neck  Total femur | Inorganic Crystal Structure Database | cross-sectional study |
| Graat-Verboom (2009)[36] | Europe | 554 (343/211) | 115 | 65.6 (0.4) | 64 ± 1.3% | 24.7 (0.2) | DXA | Whole body | Osteoporosis: T-score  < -2.35 | cross-sectional study |
| Graat-Verboom (2010)[37] | Europe | 168 (103/65) | 65 | 63.6 (9.1) | 42.1 ± 0.7% | 24.7 (4.4) | DXA | Whole body  Femur  Lumbar spine | Whole body:  osteoporosis- T-score  < − 2.35  Femur and lumbar spine: WHO | cross-sectional study |
| Graat-Verboom (2011)[38] | Europe | 255 (158/97) | 131 | 68.1 (0.6) | 44.3 ± 18.5% | 27.1 (0.3) | DXA | Lumbar spine  Hip | WHO | cross-sectional study |
| Graat-Verboom①(2012)[39] | Europe | 98 (64/34) | 49 | 69.3 | 63.4 ± 1.9% | NA | DXA | Lumbar spine  Femoral neck  Hip | WHO | cohort study |
| Graat-Verboom②(2012)[40] | Europe | 90 (54/36) | 42 | 71.7 (0.9) | 64.0 ± 8.0% | 26.5 (0.5) | DXA | Lumbar spine  Femoral neck  Hip | WHO | case-control study |
| Gupta (2014)[41] | Asia | 49 (43/6) | 32 | 60.6 (10.5) | NA | NA | DXA | NA | WHO | case-control study |
| Hattiholi (2013)[42] | Asia | 70 (45/25) | 46 | 69.2 (5.3) | 42.7 ± 16.9% | 21.4 (4.7) | DXA | Lumbar spine  Femur | WHO | cross-sectional study |
| Hattiholi (2014)[43] | Asia | 102 (64/38) | 68 | M: 66.6 (7.7)  F: 64.4 (11.5) | NA | NA | DXA | Lumbar spine | WHO | cross-sectional study |
| Incalzi (2000)[44] | Europe | 104 (68/36) | 62 | 70.9 | 40.0 ± 20.2% | 26.3 | DXA | Lumbar spine  Femoral neck | WHO | cross-sectional study |
| Jorgensen (2007)[45] | Europe | 62 (46/16) | 26 | 63.2 (5.4) | 32.6 ± 14.1% | NA | DXA | Lumbar spine  Femoral neck | WHO | cross-sectional study |
| Katsura (2002)[46] | Asia | 20 (0/20) | 10 | 72.3(1.3) | 49.9 ± 3.8% | 22 (0.8) | DXA | Whole body  Lumbar spin | Japanese Society for  Bone and Mineral  Research for  development | cross-sectional study |
| Kim (2015)[47] | Asia | 200 (180/20) | 55 | 73.1 (6) | 68.0–83.2% | 23.0 (3.3) | DXA | Lumbar spine Femoral neck | WHO | cross-sectional study |
| Lee(2015)[48] | Asia | 774 (593/181) | 124 | 66 (8) | 78.87 ± 15.02% | 23.4 (2.8) | DXA | Lumbar spine  Femur  Femur neck | WHO | cross-sectional study |
| Lee(2016)[49] | Asia | 858 (641/217) | 139 | 65.9 | NA | 23.5 | DXA | Whole body | WHO | cross-sectional study |
| Lee②(2017)[50] | Asia | 748 (575/173) | 115 | 65.8 | 76–77% | 23.4 | DXA | Lumbar spine  Hip | WHO | cross-sectional study |
| Lee①(2017)[51] | Asia | 1081 (807/274) | 191 | 64.6 | 76–80% | 23.4 | DXA | Lumbar spine  Femur  Femur neck | WHO | cross-sectional study |
| Li (2008)[52] | North America | 179 (81/98) | 118 | 59.5 (7.1) | 26.0% ± 9.8% | 25.3(5.3) | DXA | Lumbar spine  Femoral head  Hip | WHO | cross-sectional study |
| Liang (2012)[53] | Asia | 672 (583/89) | 193 | 62.5 | 58.3%±14.1% | 23.2 | DXA | Lumbar spine  Hip | WHO | cross-sectional study |
| Lin (2015)[54] | Asia | 125 (123/2) | 50 | 72.5 | 48.5% ± 19% | 23.1 | DXA | Lumbar spine  Femoral neck | WHO | cross-sectional study |
| Lin (2018)[55] | Asia | 59 (59/0) | 17 | 71.3 (7.4) | 51.0% ± 18.0% | 23.6 (4.1) | DXA | Lumbar spine  Hip | WHO | cross-sectional study |
| Liu (2015)[56] | Asia | 30 (28/2) | 19 | 70.8 | 44.3% ± 14.2% | 25.4 | DXA | Lumbar spine  Hip  Femoral neck | WHO | cross-sectional study |
| Madsen (2010)[57] | Europe | 1909 (818/1091) | 145 | 73 | NA | NA | DXA | Whole body  Lumbar spine  Hip | WHO | cross-sectional study |
| Ogura-Tomomatsu (2012）[58] | Asia | 85 (78/7) | 20 | 75 | NA | NA | DXA | Lumbar spine  Femoral head | WHO | cross-sectional study |
| Oh (2017)[59] | Asia | 137 (76/61) | 89 | 70.9 (9.3) | 57.56% ± 21.52% | 22.7 (4.9) | DXA | Lumbar spine  Femoral neck  Femur | WHO | cross-sectional study |
| Pobeha (2011)[60] | Europe | 39 (35/4) | 18 | 62.2 (7.3) | 56.4% ± 22.6% | 26.3 (6.8) | DXA | Whole body  Lumbar spine  Hip | WHO | cross-sectional study |
| Ramachandran (2016)[61] | Asia | 60 (51/9) | 21 | M: 61.2 (10.2)  F: 57.6 (7.1) | m：53.59% ± 10.6% f:60.33% ± 7.7% | NA | QUS | Calcaneus | WHO | cross-sectional study |
| Rittayamai (2012)[62] | Asia | 102 (102/0) | 32 | 71.2 (8.1) | 62.4 ± 24.1% | 21.4 (3.6) | DXA | Lumbar spine  Femoral neck | WHO | cross-sectional study |
| Romme (2012)[63] | Europe | 58 (36/22) | 27 | 65.9 | 53.7 ± 17.1% | 27 | DXA | Lumbar spine  Hip | WHO | cross-sectional study |
| Romme (2013)[64] | Europe | 151 (88/63) | 57 | 64.8 (8.8) | 45.8 ± 16.6% | 25.1 (5.4) | DXA | Lumbar spine  Hip | WHO | cross-sectional study |
| Sabit (2007)[65] | Europe | 75 (42/33) | 18 | 64.9(9.5) | 56.7 ± 21.1% | 27.6 (4.9) | DXA | Lumbar spine  Hip | WHO | cross-sectional study |
| Sakurai-Iesato (2017)[66] | Asia | 50 (50/0) | 7 | 70.4 (7.3) | 50.4 ± 17.0% | 23.2 (3.4) | DXA | Lumbar spine  Hip | WHO | cross-sectional study |
| Silva (2011)[67] | South America | 95 (62/33) | 40 | 67.3 (8.1) | 41 ± 16% | 25.8 (4.7) | DXA | Lumbar spine  Hip | WHO | cross-sectional study |
| Sim (2012)[68] | Asia | 33 (23/10) | 18 | 72 | NA | 20 | DXA | Lumbar spine | WHO | cross-sectional study |
| Tschopp (2002)[69] | Europe | 16 | 11 | 48.3 (5.3) | 32 ± 20% | 20.7 (4.8) | DXA | Lumbar spine  Femoral neck  Femoral head | WHO | cross-sectional study |
| Vaquero-Barrios (2010)[70] | Europe | 64 (51/13) | 36 | NA | NA | NA | DXA | Lumbar spine  Femoral neck | WHO | cross-sectional study |
| Vondracek (2009)[71] | North America | 23 (23/0) | 7 | 66 (8.7) | 32 ± 11% | 26.4 (6.9) | DXA | Whole body  Lumbar spine  Hip  Femoral neck | WHO | cross-sectional study |
| Vrieze (2007)[72] | Europe | 115 (62/53) | 11 | 60 (10.8) | 43.4 ± 16.9% | 26.4 (6.4) | QUS | Calcaneus | WHO | cross-sectional study |
| Wang (2014)[73] | Asia | 50 (30/20) | 38 | 80.7 (6.2) | NA | 22.1 (4.1) | DXA | Lumbar spine  Hip | WHO | case-control study |
| Watanabe (2015)[74] | Asia | 136 (136/0) | 19 | 71.6 (8.9) | 55.8 ± 25.8% | 21.5 (3.2) | DXA | Lumbar spine  Femoral neck  Hip | WHO | cross-sectional study |
| Watanabe (2017)[75] | Asia | 61 (61/0) | 23 | 70.1 (8.2) | 65.4 ± 24.1% |  | DXA | Lumbar spine  Femoral neck | WHO | cross-sectional study |
| Bolton（2011）[76] | Europe | 29 | 4 | 50–83 | 56% | 29.0（4.4） | DXA | Lumbar spine (total), hip (total), femoral neck, trochanter and intertrochanteric region. | WHO | cross-sectional study |
| EL-Gazzar（2013）[77] | Africa | 50(50/0) | 13 | 40–68 | NA | 23.86（3.75） | DXA | Lumbar spine  Hip | WHO | cross-sectional study |
| Khawar（2017）[78] | Asia | 369 | 149 | 71.44 ± 3.90 | 49.21 ± 2.94% | 21.91 ± 3.26 | DXA | Whole body  Lumbar spine  Hip | WHO | cross-sectional study |
| Kaenmuang（2023）[79] | Asia | 108(100/8) | 34 | 66.0-78.5 | 46.0-75.0 | 19.2-24.8 | DXA | Lumbar spine  Femoral neck | WHO | cohort study |
| Liao（2024）[80] | Asia | 1,297,579（128,466/146767） | 275233 | （65.62 ± 11.89） | NA | NA | NA | NA | ICD-9-CM ICD-10-CM Diagnostic code | cross-sectional study |
| Adas-Okuma（2020）[81] | South America | 91(55/36) | 27 | 66.2 ± 9.2 | NA | 27.35 ± 5.1 | DXA | Lumbar spine  Femoral neck Total hip | WHO | case-control study |
| Akyea（2019）[82] | Europe | 80,874(42,799/38,075) | 4,690 | 66.9 ± 11.0 | NA | NA | NA | NA | READ code | cohort study |
| Bitar（2021）[83] | Asia | 380（380/0） | 196 | 65.4 ± 10.04 | 56.5 ± 16 | 23.32 ± 5.43 | QUS | calcaneus | WHO | cohort study |
| CHINDAPRASIRT（2020）[84] | Asia | 107(107/0) | 38 | 69.7 ± 9.1 | NA | 23.2 ± 4.0 | DXA | Lumbar spine  Femoral neck | WHO | cross-sectional study |
| Costa（2018）[85] | South America | 121(56/65) | 58 | 67.9 ± 8.6 | NA | 26.4 ± 6.1 | DXA | Lumbar spine  Femoral neck  total femur | WHO | cross-sectional study |
| Eslaminejad（2023）[86] | Asia | 91(86/5) | 36 | 66.43 ± 9.50 | 41.94 ± 18.31 | 18.5-24.9 | DXA | Lumbar spine  Femoral neck  total femur | WHO | cross-sectional study |
| Gazzotti（2019）[87] | South America | 91(55/36) | 27 | 66.2 ± 9.2 | 55.1% ± 21.4% | 27.3 ± 5.2 | DXA | Lumbar spine  Total femur Proximal femur | WHO | cross-sectional study |
| Graumam（2018）[88] | South America | 99（46/53） | 40 | 65.1 ± 8.9 | 51.3% ± 17.3% | 26.4 ± 6.1 | DXA | Lumbar spine  Femoral neck  total femur | ISCD | cross-sectional study |
| Graumam（2026）[89] | South America | 40(19/27) | 12 | 69.0 ± 7.5 | NA | 25.95 ± 5.21 | DXA | Lumbar spine  Femoral neck  total femur | ISCD | cohort study |
| Hu（2024）[90] | Asia | 81(46/35) | 37 | 50 - 80 | NA | NA | DXA | Lumbar spine Thoracolumbar spine | WHO | cross-sectional study |
| Bağcıer（2019）[91] | Asia | 88（88/0） | 44 | 55–84 | NA | NA | DXA | Lumbar spine  Femoral neck | WHO | cross-sectional study |
| ISLAM（2018）[92] | Asia | 30(28/2) | 17 | 63.47 ± 8.45 | 44.45% ± 14.64% | 23.09 ± 4.82 | DXA | Lumbar spine  Femoral neck | WHO | cross-sectional study |
| Kahnert（2018）[93] | Europe | 2,228(1361/867) | 352 | 65.1 ± 8.4 | 52.8% ± 18.5% | 26.7 ± 5.2 | NA | NA | NA | cross-sectional study |
| Kahnert（2021）[94] | Europe | 2,222(1359/863) | 351 | 65.0 ± 8.4 | 52.8% ± 18.4% | 26.6 ± 5.2 | NA | NA | NA | cross-sectional study |
| Kameyama（2019）[95] | Asia | 111(105/6) | 8 | 72.5 ± 8.1 | 68.3% ± 20.3% | 23.5 ± 3.023.5 ± 3.0 | DXA | lumbar spine  both femurs | WHO | cohort study |
| Lee（2021）[96] | Asia | 52,652（6596/6567） | 13,163 | 30-70 | NA | NA | NA |  | WHO | cohort study |
| Yormaz（2020）[97] | Asia | 94(94/0) | 35 | 67.57 ± 7.42 | NA | 23.47 ± 4.45 | DXA | Lumbar spine  Femoral neck | WHO | cross-sectional study |
| Malaise（2023）[98] | Europe | 90(56/34) | 23 | 63.4 ± 10.1 | NA | 24.1 ± 5.2 | DXA | lumbar spine  left total hip  Left femoral neck | WHO | cross-sectional study |
| Nasrallah（2019）[99] | Africa | 50(35/15) | 16 | 59.04 ± 7.14 | NA | 21.86 ± 3.75 | DXA | Lumbar spine  Femoral neck  distal radius and ulna | WHO | case-control study |
| Ozcakir（2020）[100] | Asia | 30(25/5) | 12 | 41-79 | 51.85% ± 14.60% | 26.46 ± 3.16 | DXA | Lumbar spine   left hip | WHO | cross-sectional study |
| Trigueros（2019）[101] | Europe | 1,610(1324/286) | 286 | f:63.4 ± 10.2 m 67.4 ± 9.4 | f 59.6% ± 14.0 m 53.8% ± 14.2% | f 27.4 ± 5.2 28.0 ± 4.6 | NA | NA | NA | cross-sectional study |
| Trudzinski（2024）[102] | Europe | 418(241/177) | 50 | 64±5 | 46.9 - 77.6 % | 26.5 | CT | TH12 Thoracic vertebrae |  | cohort study |
| Vikjord（2019）[103] | Europe | 3,239(1,683/1556) | 444 | 40 - 85 | 64.4% (±19.6%) | 26.7 ± 4.9 | DXA | total hip  distal forearm | WHO | cohort study |
| Yazıcı（2021）[104] | Asia | 95(80/15) | 39 | 66.46 ± 8.07 | 52.47% ± 15.94% | 25.31 ± 4.59 | DXA | Lumbar spine  Femoral neck  Femur total | WHO | cross-sectional study |

**Supplementary Table S2:** Newcastle-Ottawa Quality Assessment for Cohort and Case-Control Studies

Note: A study can be given a maximum of one star for each numbered item within the Selection and Outcome categories. A

maximum of two stars can be given for Comparability.

**Selection**

1) Representativeness of the exposed cohort

a) Truly representative ***(one star)***

b) Somewhat representative ***(one star)***

c) Selected group

d) No description of the derivation of the cohort

2) Selection of the non-exposed cohort

a) Drawn from the same community as the exposed cohort ***(one star)***

b) Drawn from a different source

c) No description of the derivation of the non exposed cohort

3) Ascertainment of exposure

a) Secure record (e.g., surgical record) ***(one star)***

b) Structured interview ***(one star)***

c) Written self report

d) No description

e) Other

4) Demonstration that outcome of interest was not present at start of study

a) Yes ***(one star)***

b) No

**Comparability**

1) Comparability of cohorts on the basis of the design or analysis controlled for confounders

a) The study controls for age, sex and marital status ***(one star)***

b) Study controls for other factors (list) _________________________________ ***(one star)***

c) Cohorts are not comparable on the basis of the design or analysis controlled for confounders

**Outcome**

1) Assessment of outcome

a) Independent blind assessment ***(one star)***

b) Record linkage ***(one star)***

c) Self report

d) No description

e) Other

2) Was follow-up long enough for outcomes to occur

a) Yes ***(one star)***

b) No

Indicate the median duration of follow-up and a brief rationale for the assessment above:____________________

3) Adequacy of follow-up of cohorts

a) Complete follow up- all subject accounted for ***(one star)***

b) Subjects lost to follow up unlikely to introduce bias- number lost less than or equal to 20% or description of those lost

suggested no different from those followed. ***(one star)***

c) Follow up rate less than 80% and no description of those lost

d) No statement

E-17Thresholds for converting the Newcastle-Ottawa scales to AHRQ standards (good, fair, and

poor):

**Good quality:** 3 or 4 stars in selection domain AND 1 or 2 stars in comparability domain AND 2

or 3 stars in outcome/exposure domain

**Fair quality:** 2 stars in selection domain AND 1 or 2 stars in comparability domain AND 2 or 3

stars in outcome/exposure domain

**Poor quality:** 0 or 1 star in selection domain OR 0 stars in comparability domain OR 0 or 1 stars

in outcome/exposure domain

The Newcastle-Ottawa Quality Assessment scale to evalute risk of bias in three domains for cohort study and Case-Control Study.

| Studies | Selection | Comparability | Outcome | Total score |
| --- | --- | --- | --- | --- |
| Graat-Verboom①(2012)[39] | 4 | 2 | 2 | 8 |
| Graat-Verboom②(2012)[40] | 3 | 2 | 3 | 8 |
| Gupta (2014)[41] | 2 | 0 | 1 | 3 |
| Wang (2014)[73] | 3 | 1 | 2 | 6 |
| Kaenmuang(2023)[79] | 4 | 2 | 3 | 9 |
| Adas-Okuma(2019)[81] | 4 | 1 | 3 | 8 |
| Akyea(2019)[82] | 3 | 2 | 3 | 8 |
| Bitar(2021)[83] | 2 | 2 | 2 | 6 |
| Graumam(2025)[89] | 1 | 2 | 3 | 6 |
| Kameyama(2019)[95] | 4 | 2 | 3 | 9 |
| Lee(2020)[96] | 4 | 2 | 3 | 9 |
| Nasrallah（2019）[99] | 4 | 1 | 2 | 7 |
| Trudzinski(2024)[102] | 4 | 2 | 3 | 9 |
| Vikjord(2019)[103] | 4 | 2 | 3 | 9 |

**Supplementary Table S3: Cross-Sectional/Prevalence Study Quality**

| Item | Yes | No | Unclear |
| --- | --- | --- | --- |
| 1) Define the source of information (survey, record review) |  |  |  |
| 2) List inclusion and exclusion criteria for exposed and unexposed subjects (cases and controls) or refer to previous publications |  |  |  |
| 3) Indicate time period used for identifying patients |  |  |  |
| 4) Indicate whether or not subjects were consecutive if not population-based |  |  |  |
| 5) Indicate if evaluators of subjective components of study were masked to other aspects of the status of the participants |  |  |  |
| 6) Describe any assessments undertaken for quality assurance purposes (e.g., test/retest of primary outcome measurements) |  |  |  |
| 7) Explain any patient exclusions from analysis |  |  |  |
| 8) Describe how confounding was assessed and/or controlled. |  |  |  |
| 9) If applicable, explain how missing data were handled in the analysis |  |  |  |
| 10) Summarize patient response rates and completeness of data collection |  |  |  |
| 11) Clarify what follow-up, if any, was expected and the percentage of patients for which incomplete data or follow-up was obtained |  |  |  |
|  |  |  |  |

| First Author (Year) | ① | ② | ③ | ④ | ⑤ | ⑥ | ⑦ | ⑧ | ⑨ | ⑩ | ⑪ | Total score |
| --- | --- | --- | --- | --- | --- | --- | --- | --- | --- | --- | --- | --- |
| Abbasi (2016)[18] | Y | Y | Y | NC | N | N | N | N | N | N | Y | 4 |
| Arenas-de Larriva  (2010)[19] | Y | Y | Y | Y | N | N | N | N | N | N | Y | 5 |
| Bhattacharyya (2011)[20] | Y | Y | Y | NC | N | N | N | N | N | N | Y | 4 |
| Bolton (2004)[21] | Y | Y | N | NC | Y | Y | Y | Y | N | N | Y | 7 |
| Bolton (2008)[22] | Y | Y | Y | NC | N | Y | Y | Y | N | N | N | 6 |
| Bolton (2009)[23] | Y | Y | Y | NC | Y | Y | Y | Y | N | N | N | 7 |
| Bon (2010)[24] | Y | Y | Y | N | N | Y | N | Y | Y | N | N | 6 |
| Chubachi (2014)[25] | Y | Y | Y | NC | NC | Y | Y | Y | NC | NC | N | 6 |
| Coin (2010)[26] | Y | Y | N | Y | NC | NC | N | Y | NC | NC | N | 4 |
| Dimai (2001)[27] | Y | Y | Y | NC | N | Y | Y | Y | NC | NC | N | 6 |
| Dubois (2002)[28] | Y | N | Y | Y | N | Y | N | Y | NC | N | N | 5 |
| Duckers(2011)[29] | Y | Y | N | NC | N | Y | Y | Y | NC | Y | N | 6 |
| Xu（2020）[30] | Y | Y | Y | N | N | N | N | Y | N | N | N | 4 |
| Fouda (2017)[31] | Y | Y | Y | Y | NC | Y | Y | Y | NC | Y | N | 8 |
| Fountoulis (2012)[32] | Y | Y | N | NC | N | Y | Y | Y | NC | Y | N | 6 |
| Fountoulis (2016[33] | Y | Y | Y | NC | Y | Y | Y | Y | NC | N | N | 7 |
| Franco (2009)[34] | Y | Y | Y | Y | N | N | Y | Y | NC | N | N | 6 |
| Gonçalves (2017)[35] | Y | Y | Y | NC | NC | Y | Y | Y | NC | N | N | 6 |
| Graat-Verboom (2009)[36] | Y | Y | Y | Y | N | Y | Y | Y | NC | N | N | 7 |
| Graat-Verboom (2010)[37] | Y | Y | Y | Y | N | N | Y | Y | NC | N | N | 6 |
| Graat-Verboom (2011)[38] | Y | Y | Y | Y | Y | N | Y | Y | NC | N | N | 7 |
| Hattiholi (2013)[42] | Y | Y | Y | NC | N | N | Y | Y | NC | N | N | 5 |
| Hattiholi (2014)[43] | Y | Y | Y | NC | N | N | Y | Y | NC | N | N | 5 |
| Incalzi (2000)[44] | Y | Y | Y | Y | NC | Y | Y | Y | N | NC | N | 7 |
| Jorgensen (2007)[45] | Y | Y | Y | Y | Y | Y | Y | N | Y | Y | N | 9 |
| Katsura (2002)[46] | Y | Y | N | NC | Y | Y | Y | N | N | NC | N | 5 |
| Kim (2015)[47] | Y | Y | Y | N | NC | Y | Y | Y | N | N | N | 6 |
| Lee (2015)[48] | Y | Y | Y | NC | Y | Y | Y | Y | N | Y | N | 8 |
| Lee (2016)[49] | Y | Y | Y | NC | Y | Y | Y | Y | N | Y | N | 8 |
| Lee②(2017)[50] | Y | Y | Y | N | NC | Y | Y | Y | N | NC | N | 6 |
| Lee①(2017)[51] | Y | Y | Y | Y | N | Y | Y | Y | N | N | N | 7 |
| Li (2008)[52] | Y | Y | Y | Y | NC | Y | Y | Y | N | NC | N | 7 |
| Liang (2012)[53] | Y | Y | Y | N | NC | N | N | Y | N | N | N | 4 |
| Lin (2015)[54] | Y | Y | Y | Y | NC | Y | Y | Y | Y | Y | N | 9 |
| Lin (2018)[55] | Y | Y | Y | NC | NC | Y | Y | Y | N | NC | N | 6 |
| Liu (2015)[56] | Y | Y | N | NC | NC | Y | Y | Y | N | NC | N | 5 |
| Madsen (2010)[57] | Y | Y | Y | Y | Y | Y | Y | Y | N | Y | Y | 10 |
| Ogura-Tomomatsu  (2012）[58] | Y | Y | N | NC | Y | N | Y | Y | N | N | N | 5 |
| Oh (2017)[59] | Y | Y | Y | N | NC | N | Y | Y | N | N | N | 5 |
| Pobeha (2011)[60] | Y | Y | N | N | NC | N | N | Y | N | N | N | 3 |
| Ramachandran (2016)[61] | Y | Y | Y | Y | NC | Y | N | N | N | N | N | 5 |
| Rittayamai (2012)[62] | Y | Y | Y | N | NC | N | N | Y | N | N | N | 4 |
| Romme (2012)[63] | Y | Y | Y | N | Y | Y | Y | N | N | N | N | 6 |
| Romme (2013)[64] | Y | Y | Y | N | NC | N | Y | Y | N | N | N | 5 |
| Sabit (2007)[65] | Y | Y | N | N | NC | N | N | Y | N | N | N | 3 |
| Sakurai-Iesato (2017)[66] | Y | Y | Y | N | NC | N | Y | Y | N | N | N | 5 |
| Silva (2011)[67] | Y | Y | Y | Y | NC | Y | Y | Y | N | Y | N | 8 |
| Sim (2012)[68] | Y | Y | Y | NC | N | NC | Y | Y | N | N | N | 5 |
| Tschopp (2002)[69] | Y | Y | Y | NC | N | NC | Y | Y | N | Y | N | 5 |
| Vaquero-Barrios (2010)[70] | Y | Y | Y | NC | N | N | Y | N | N | NC | N | 4 |
| Vondracek (2009)[71] | Y | Y | N | NC | N | Y | Y | Y | N | Y | N | 6 |
| Vrieze (2007)[72] | Y | Y | Y | Y | N | Y | N | Y | N | Y | Y | 8 |
| Watanabe (2015)[74] | Y | Y | Y | N | N | N | Y | Y | N | N | N | 5 |
| Watanabe (2018)[75] | Y | Y | Y | NC | N | NC | Y | Y | N | N | N | 5 |
| Bolton（2011）[76] | Y | Y | N | NC | Y | Y | Y | Y | NC | NC | N | 6 |
| EL-Gazzar（2013）[77] | Y | Y | Y | NC | NC | N | N | N | NC | N | N | 4 |
| Khawar（2017）[78] | Y | Y | Y | Y | NC | Y | N | N | NC | NC | N | 5 |
| Liao（2024）[80] | Y | Y | Y | N | N | Y | Y | Y | Y | N | N | 7 |
| CHINDAPRASIRT（2020）[84] | Y | Y | Y | N | N | N | Y | Y | Y | N | N | 6 |
| Costa（2018）[85] | Y | Y | Y | Y | N | Y | Y | N | Y | Y | N | 8 |
| Eslaminejad （2023）[86] | Y | Y | Y | N | N | N | N | Y | N | N | N | 4 |
| Gazzotti（2018）[87] | Y | Y | N | Y | N | Y | Y | Y | Y | Y | N | 8 |
| Graumam（2018）[88] | Y | Y | Y | Y | Y | Y | Y | Y | Y | Y | N | 10 |
| Hu（2024）[90] | Y | Y | Y | N | N | N | Y | Y | N | N | N | 5 |
| Bağcıer（2019）[91] | Y | Y | Y | NC | N | N | N | N | N | N | N | 3 |
| ISLAM （2018）[92] | Y | Y | Y | N | N | N | N | N | N | N | N | 3 |
| Kahnert（2018）[93] | Y | Y | Y | NC | N | Y | Y | Y | Y | Y | N | 8 |
| Kahnert（2021）[94] | Y | Y | Y | NC | N | Y | Y | Y | Y | Y | N | 8 |
| Yormaz(2019)[97] | Y | Y | Y | NC | N | Y | N | Y | N | N | N | 5 |
| Malaise （2023）[98] | Y | Y | N | Y | NC | Y | Y | Y | Y | Y | N | 8 |
| Ozcakir（2020）[100] | Y | Y | Y | N | N | N | Y | N | N | N | N | 4 |
| Trigueros（2019）[101] | Y | Y | Y | Y | N | Y | Y | N | N | Y | N | 7 |
| Yazıcı(2021)[104] | Y | Y | Y | NC | N | N | Y | N | Y | Y | N | 6 |

**Supplementary Figure S1:** Forest Plot:The overall prevalence of COPD with OP and the corresponding subgroup analysis by continent are presented.

**Supplementary Figure S2:** Forest Plot:The prevalence of COPD with OP stratified by Study design is presented.

**Supplementary Figure S3:** Forest Plot:The prevalence of COPD with OP stratified by BMI is presented.

**Supplementary Figure S4:** Forest Plot:The prevalence of COPD with OP stratified by Age is presented.

**Supplementary Figure S5:** Forest Plot:The prevalence of COPD with OP stratified by BMD assessment method is presented.

**Supplementary Figure S6:** Scatter plot with regression line of effect size (ES) by number.

**Supplementary Figure S7:**Scatter plot with regression line of effect size (ES) by continent.

**Supplementary Figure S8:**Scatter plot with regression line of effect size (ES) by year.

**Supplementary Figure S9:**Scatter plot with regression line of effect size (ES) by Literature quality score.

**Supplementary Figure S10:**Scatter plot with regression line of effect size (ES) by Study design.

**Supplementary Figure S11:**Scatter plot with regression line of effect size (ES) by Mean participant age.

**Supplementary Figure S12:**Scatter plot with regression line of effect size (ES) by BMD assessment method.

**Supplementary Figure S13:** Forest Plot:Risk factors for COPD with OP stratified by Sex

**Supplementary Figure S14:** Sensitivity analysis:Risk factors for COPD with OP stratified by Sex

**Supplementary Figure S15:** Funnel plot:Risk factors for COPD with OP stratified by Sex

**Supplementary Figure S16:** Forest Plot:Risk factors for COPD with OP stratified by BMI

**Supplementary Figure S17:** Sensitivity analysis:Risk factors for COPD with OP stratified by BMI

**Supplementary Figure S18:** Funnel plot:Risk factors for COPD with OP stratified by BMI

**Supplementary Figure S19:** Forest Plot:Risk factors for COPD with OP stratified by Corticosteroids

**Supplementary Figure S20:** Sensitivity analysis:Risk factors for COPD with OP stratified by Corticosteroids

**Supplementary Figure S21:** Funnel plot:Risk factors for COPD with OP stratified by Corticosteroids

**Supplementary Figure S22:** Forest Plot:Risk factors for COPD with OP stratified by FEV1% predicted

**Supplementary Figure S23:** Funnel plot:Risk factors for COPD with OP stratified by FEV1% predicted

**Supplementary Figure S24:** Forest Plot:Risk factors for COPD with OP stratified by Age

**Supplementary Figure S25:** Sensitivity analysis:Risk factors for COPD with OP stratified by Age

**Supplementary Figure S26:** Funnel plot:Risk factors for COPD with OP stratified by Age
